# Maintenance of complex I and its supercomplexes by NDUF-11 is essential for mitochondrial structure, function and health

**DOI:** 10.1242/jcs.258399

**Published:** 2021-07-09

**Authors:** Amber Knapp-Wilson, Gonçalo C. Pereira, Emma Buzzard, Holly C. Ford, Andrew Richardson, Robin A. Corey, Chris Neal, Paul Verkade, Andrew P. Halestrap, Vicki A. M. Gold, Patricia E. Kuwabara, Ian Collinson

**Affiliations:** 1 School of Biochemistry, University of Bristol, Bristol BS8 1TD, UK; 2 Living Systems Institute, Stocker Road, University of Exeter, Exeter EX4 4QD, UK; 3 College of Life and Environmental Sciences, Geoffrey Pope Building, University of Exeter, Stocker Road, Exeter EX4 4QD, UK; 4 Department of Biochemistry, University of Oxford, Oxford OX1 3QU, UK; 5 Wolfson Bioimaging Facility, Faculty of Life Sciences, University of Bristol, Bristol BS8 1TD, UK

**Keywords:** *Caenorhabditis elegans*, Worm, Mitochondria, Respiration, Electron transfer chain, Supercomplexes, Respirasome, Mitochondrial ultrastructure, Cryo-electron tomography, NDUF-11

## Abstract

Mitochondrial supercomplexes form around a conserved core of monomeric complex I and dimeric complex III; wherein a subunit of the former, NDUFA11, is conspicuously situated at the interface. We identified *nduf-11* (*B0491.5*) as encoding the *Caenorhabditis elegans* homologue of NDUFA11. Animals homozygous for a CRISPR-Cas9-generated knockout allele of *nduf-11* arrested at the second larval (L2) development stage. Reducing (but not eliminating) expression using RNAi allowed development to adulthood, enabling characterisation of the consequences: destabilisation of complex I and its supercomplexes and perturbation of respiratory function. The loss of NADH dehydrogenase activity was compensated by enhanced complex II activity, with the potential for detrimental reactive oxygen species (ROS) production. Cryo-electron tomography highlighted aberrant morphology of cristae and widening of both cristae junctions and the intermembrane space. The requirement of NDUF-11 for balanced respiration, mitochondrial morphology and development presumably arises due to its involvement in complex I and supercomplex maintenance. This highlights the importance of respiratory complex integrity for health and the potential for its perturbation to cause mitochondrial disease.

This article has an associated First Person interview with Amber Knapp-Wilson, joint first author of the paper.

## Abstract (precis)

**Summary:** Destabilisation of complex I and its supercomplexes by removal of the assembly factor NDUF-11 severely affects balanced respiration, mitochondrial morphology and, ultimately, whole animal physiology.

## INTRODUCTION

Mitochondria are dynamic organelles operating as hubs for energy production, metabolic and biosynthetic pathways and Ca^2+^ homeostasis. Consequently, they are intrinsically associated with oxidative stress, proteostasis and cell signalling responsible for maintaining cellular and organismal health, as well as for survival (Annesley and Fisher, 2019; Bravo-Sagua et al., 2017; Hekimi et al., 2016; Moehle et al., 2019). They generate ATP by oxidative phosphorylation (OXPHOS) to power cellular activities (Mitchell, 2011; Nicholls and Ferguson, 2013). This is achieved by four large multimeric electron transfer complexes (complexes I–IV) and the ATP synthase of the inner mitochondrial membrane (IMM). The complexes I–IV (CI–CIV) form a continuous electron transfer chain (ETC), orchestrating the step-wise transfer of electrons from NADH (complex I, NADH:ubiquinone oxidoreductase) and FADH_2_ (complex II, succinate:ubiquinone oxidoreductase), via complex III (cytochrome *bc_1_*) to molecular oxygen at complex IV (cytochrome *c* oxidase). This flow of electrons down an electro-chemical redox potential liberates energy, which is conserved by proton pumping across the IMM from the matrix to the intermembrane space (IMS). This electro-chemical gradient of protons – the proton-motive force (PMF) – is used to power the ATP synthase.

**Fig. 1.**
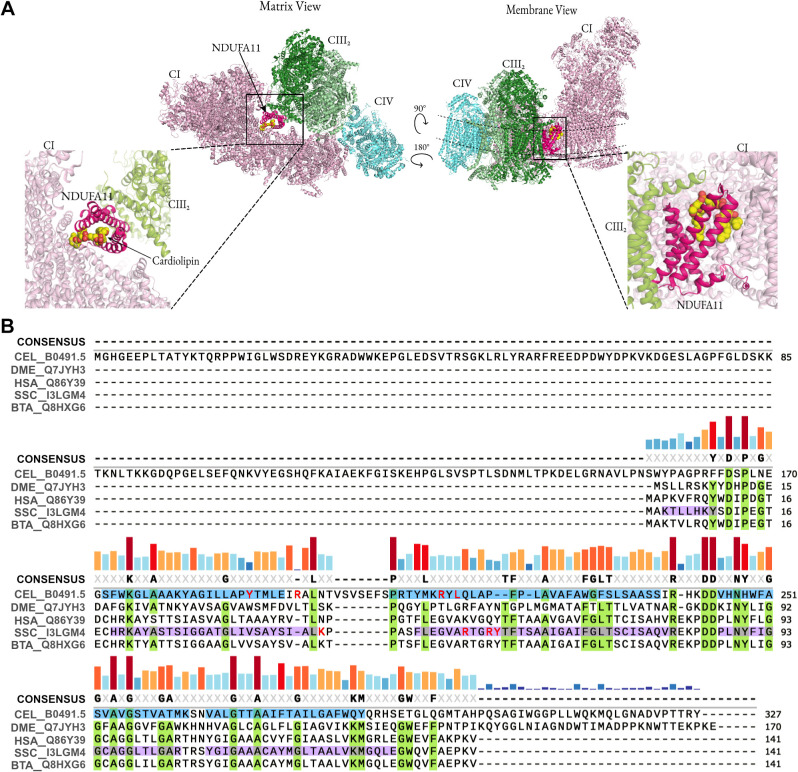
**Mitochondrial complexes and supercomplexes with subunit NDUFA11.** (A) Cryo-EM structure of the human respirasome SCI:III_2_:IV (PDB:5XTH; Guo et al., 2016) viewed from the matrix (top left) and membrane (top right) rotated about the membrane axis. The boxed area highlights NDUFA11 and cardiolipin, and the dashed lines represent the position of the inner membrane (matrix above and IMS below). CI, light pink; CIII_2_ (dimer of CIII), green; CIV, blue; NDUFA11, magenta; cardiolipin, yellow. (B) Multiple sequence alignment of NDUFA11 homologues with NDUF-11 (CEL-B0491.5), generated using Clustal Omega (Madeira et al., 2019) and graphically handled in SnapGene. Amino acids highlighted in lilac represent TMH regions based on porcine NDUFA11 (PDB:5GUP; Wu et al., 2016), while those in cyan are predicted CEL-B0491.5 TMH regions determined by JPred4 (Drozdetskiy et al., 2015). Conserved residues across entries are highlighted in light green. The sequence conservation is shown by the coloured vertical bars, and a threshold of >75% was used for the consensus. Residues important for cardiolipin binding are highlighted in red. Species and Uniprot accession are indicated for each sequence except CEL-B0491.5, which has Uniprot accession Q17512 (CEL, *C. elegans*; DME, *Drosophila melanogaster*; HSA, *Homo sapiens*; SSC, *Sus scrofa*; BTA, *Bos taurus*).

The respiratory complexes can form functional higher-order assemblies with defined stoichiometries (Davies et al., 2018 and references therein). These supercomplexes, also known as *‘*respirasomes’, were first observed using non-denaturing blue native-polyacrylamide gel electrophoresis (BN-PAGE) (Schagger and Pfeiffer, 2000). The mammalian supercomplexes, visualised using cryo-electron microscopy (cryo-EM), consist of monomeric CI associated with dimeric CIII and a single CIV (SCI_1_:III_2_:IV_1_; Fig. 1A) (Gu et al., 2016; Letts et al., 2016; Sousa et al., 2016). The acquisition of such a supercomplex structure has lent credibility to the existence of discrete entities for the containment of the entire ETC; in contrast to the classical view of discontinuous electron transfer complexes connected by mobile electron carriers. Various supercomplexes, consisting of a conserved core of SCI_1_:III_2_, have been visualised using cryo-electron tomography (cryo-ET) *in situ –* in the flat surface of cristae membranes of yeast, plants and mammals – with slight variations observed in the position and stoichiometry of CIV (Davies et al., 2018, 2011). This common architecture of SCI_1_:III_2_ is highly suggestive of an important functional and/or structural role of the supercomplexes. However, despite the evidence supporting the existence of structurally conserved respiratory supercomplexes, the significance of these assemblies remains an open question.

We set out to address this problem by exploiting the genetically tractable multicellular model organism *Caenorhabditis elegans*, wherein we identified and examined the role of the homologue of mammalian NDUFA11 – a supernumerary subunit of complex I. NDUFA11 is an integral membrane protein that is positioned (amongst others) at the interface between complex I and complex III and is posited to support a critical interaction within the respiratory supercomplex (Fig. 1A) (Gu et al., 2016; Letts et al., 2016, 2019; Sousa et al., 2016). NDUFA11 has previously been implicated in complex I assembly (Andrews et al., 2013; Blaza et al., 2018; Nehls et al., 1992) and its perturbed expression – caused by a faulty splicing – results in fatal infantile lactic acidemia, encephalocardiomyopathy and late-onset myopathy (Berger et al., 2008; Peverelli et al., 2019). It is unclear whether loss of NDUFA11 levels also results in supercomplex instability, or indeed whether this higher-order destabilisation accounts for thedocumented, or indeed any other, mitochondrial disease.

Our study confirms the importance of NDUFA11 for development to adulthood and for the assembly and maintenance of complex I and the respiratory supercomplexes. Depletion of the *C. elegans* NDUFA11 homologue led to destabilisation of respiratory complex I and its supercomplex; this in turn enabled us to assess the importance of their integrity for mitochondrial function and morphology, as well as the consequences of the effects of this destabilisation for whole animal physiology and health.

## RESULTS

### Identification of the *C. elegans NDUFA11* homologue

Mammalian NDUFA11 is a supernumerary subunit of complex I that is located in the membrane arm and in contact with complex III within the respiratory supercomplex (Fig. 1A; Fig. S1A). Interestingly, it is also associated with a tightly bound phospholipid cardiolipin (CL), situated on the matrix side of the IMM (Fig. 1A; Fig. S1A,B). It is composed of a bundle of four transmembrane helices (TMHs) flanked by a short N-terminal helix and a C-terminal loop region. The *C. elegans B0491.5* gene was initially identified as encoding an *NDUFA11* homologue by PSI-BLAST using the gene product sequence of the *Drosophila* counterpart of NDUFA11 (ND-B14.7) as the query (E-value=2×10^-6^; identity=26%). A multiple sequence alignment of mammalian and *Drosophila* NDUFA11 proteins with worm B0491.5 highlighted the conservation across homologues of membrane topology and CL-binding residues (Fig. 1B). *C. elegans* B0491.5 also has a worm-specific N-terminal extension (Fig. 1B).

Given the availability of several high-resolution structures of respiratory complexes containing NDUFA11 (Gu et al., 2016; Letts et al., 2016; Sousa et al., 2016), the structural conservation of worm B0491.5 was explored by generating a homology model based on the cryo-EM structure of porcine NDUFA11 (PDB:5GUP; Wu et al., 2016) using Modeller (Webb and Sali, 2016) and by molecular dynamics simulations (for details refer to Materials and Methods; Fig. S1B). The derived structure was stable (Fig. S1C) and showed the conservation of predicted features, including TMH regions and a pocket to support CL binding (Fig. S1D, orange, right). As mentioned above, B0491.5 has a considerable (155 amino acids) N-terminus; however, we omitted both the N- and C-termini from our homology model as these regions have very low predicted secondary structure and no predicted transmembrane regions, suggesting that they are likely extra-membrane loop regions. Taken together, the sequence analysis and homology modelling confirm the classification of *C. elegan*s B0491.5, hereafter referred to as NDUF-11 (encoded by *nduf-11*), as an NDUFA11 homologue.

### Reduction of *nduf-11* expression causes growth arrest and lifespan extension

**Fig. 2.**
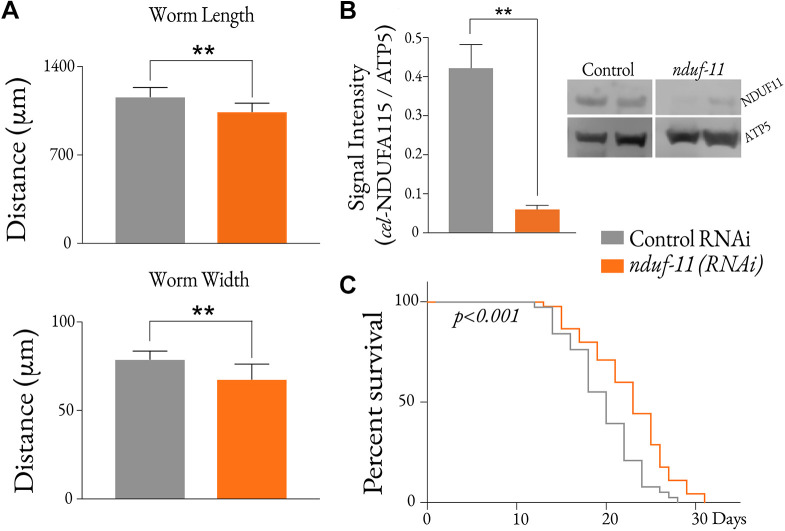
**Characterisation of *nduf-11(RNAi)­*-treated worms.** (A) Morphometric analysis of *nduf-11(RNAi)* adult worms. *n*=11 (wild type) and *n*=8 *nduf-11(RNAi)* adults were analysed. Data shown as mean±s.e.m. **P<0.01 (Student's *t*-test). (B) Reduction of NDUF-11 protein in mitochondria after RNAi treatment. A total of 50 µg of isolated mitochondria was loaded per lane. Data shown as mean±s.e.m. of *n*=5. Representative blots are shown on the right. ATP-5 was used as a loading control. **P<0.01 (Student's *t*-test). (C) *nduf-11(RNAi)* is responsible for a lifespan extension*.* Wild-type, *n*=45; *nduf-11(RNAi)*, *n*=38. Kaplan–Meier survival statistics are shown.

An *nduf-11(cr51)* deletion allele designed to abrogate protein-coding potential was obtained using CRISPR-Cas9 gene editing. Animals homozygous for *nduf-11(cr51)* – i.e. devoid of protein – arrested at the second larval stage (L2) of development, a lethal phenotype commonly displayed by animals carrying mutations in nuclear genes encoding mitochondrial proteins (Lemire, 2005). Because, as we have shown, *nduf-11(cr51)* homozygotes display an early larval arrest, it was not possible to obtain sufficient biomass for biochemical analysis. Instead, viable *C. elegans* with reduced *nduf-11* levels were recovered using feeding RNAi to enable the bulk isolation of mitochondria. Adults continuously fed with *nduf-11(RNAi)* were smaller and thinner, and they produced fewer progeny than control mock RNAi-treated N2 adults (Fig. 2A; Table S1). However, similar to *nudf-11*-knockout mutants, the progeny produced by these RNAi-treated adults failed to develop into fecund adults and arrested at the L2 stage. Western blot quantification estimated that RNAi led to an ∼83% reduction in NDUF-11 protein levels (Fig. 2B). Continuous application of *nduf-11(RNAi)* also led to an extension in lifespan when compared to that of mock RNAi-treated control animals (Fig. 2C), in agreement with a previous observation (Chen et al., 2007). These analyses confirm that *nduf-11(RNAi)* treatment is effective in reducing the corresponding protein levels, while allowing worms to develop into fecund adults.

### Analysis of the mitochondrial and cytosolic proteome following NDUF-11 depletion by RNAi

The underlying cause of the lethality of the *nduf-11* knockout (complete loss of expression) was assessed through an examination of the less severe phenotype observed upon reduction of *nduf-11* expression by RNAi treatment. This analysis began with an investigation of the corresponding changes in the mitochondrial and cytosolic proteomes. For this purpose, mitochondrial and cytosolic fractions were collected from whole worms (see Materials and Methods for details); the latter fraction being, as much as possible, devoid of nuclear, plasma membrane and other endosomal systems.

**Fig. 3.**
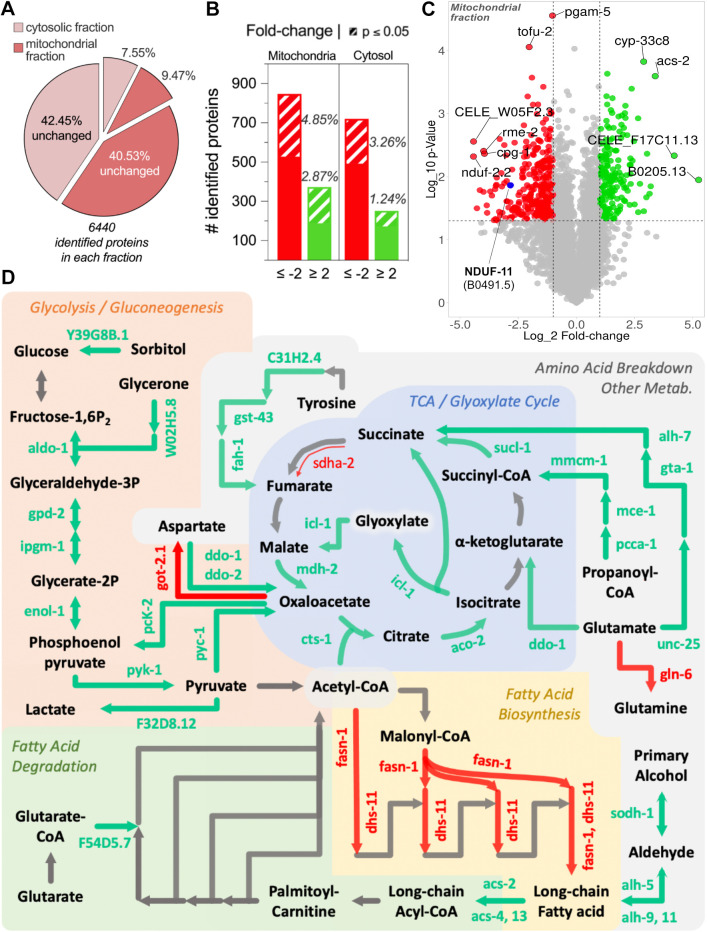
**Mass spectrometry analysis of the cytosolic and isolated mitochondrial fractions from control and *nduf-11(RNAi)* groups.** (A) The exploded slices of the pie chart indicate the proportion of proteins that were up- or down-regulated in cytosolic or mitochondrial fractions. (B) Bar chart showing the number of proteins that were either upregulated (green) or downregulated (red) in each fraction, using a fold-change threshold of 2. The percentage of the total identified proteins that were significantly up- or down-regulated is shown*. n*=3. (C) Volcano-plot showing protein expression changes in isolated mitochondria after RNAi treatment. NDUF-11 is highlighted in blue on the plot. Proteins significantly upregulated are shown in green, while those significantly downregulated are shown in red. Proteins with levels similar to control levels are depicted in grey. Dashed lines indicate thresholds for significant changes in protein level. The top ten most significant hits are labelled, and their human orthologues are shown in Table S2. *n*=3. (D) Summary of the major changes in several metabolic pathways after RNAi treatment. The significantly up- and down-regulated proteins from the cytosolic and mitochondrial fractions, as shown in A and B, were used for KEGG analysis. The majority of changes were observed in the metabolic pathways depicted here in boxes with different backgrounds (glycolysis–gluconeogenesis, amino acid breakdown, fatty acid biosynthesis and breakdown, tricarboxylic acid cycle and glyoxylate cycle). Green arrows represent the activity of proteins that were upregulated upon *nduf-11(RNAi)* silencing and, conversely, red arrows represent activity of downregulated proteins. Grey arrows represent metabolic steps in the depicted pathway regulated by proteins whose levels remained unchanged. Thin arrow represents germline-specific isoforms.

Quantitative mass spectrometry comparing the proteomes of animals with native and depleted levels of NDUF-11 protein identified 6440 proteins in each fraction, of which less than 10% were significantly up- or down-regulated (Fig. 3A). Of the affected proteins, the majority were downregulated upon NDUF-11 depletion, with 4.85% and 3.26% attaining statistical significance for the mitochondrial and cytosolic fractions, respectively (Fig. 3B). In contrast, only 2.87% and 1.24% were upregulated in the corresponding mitochondrial and cytosolic fractions, respectively (Fig. 3B). As expected, the data confirmed that NDUF-11 was downregulated to ∼20% of wild-type levels (Fig. 3C, blue dot), similar to the value estimated by western blotting (Fig. 2B). The remaining complex I subunits were downregulated by ∼50% (Fig. S2), suggesting that NDUF-11 is indeed important for complex I integrity, as previously documented (Andrews et al., 2013). Conversely, other respiratory complex subunits, namely complexes III, IV and V, were marginally upregulated, albeit not above a statistically significant threshold (Fig. S2). Crucially, for the present investigation (see below), the four subunits of complex II, SDHA-1, SDHB-1, MEV-1 and SDHD-1 (Fig. S2), were upregulated by 1.73- (*P*=0.0225), 1.39- (*P*=0.0188), 1.24- (*P*=0.1209) and 1.28- (*P*=0.009) fold, respectively.

In addition to changes in the levels of the ETC constituents, we observed downregulation of proteins responsible for fatty acid biosynthesis, and an upregulation in the rate-limiting enzyme of β-oxidation, acyl-CoA synthetase (ACS; encoded by *acs-2* in *C. elegans*), indicative of an upregulation of the fatty acid catabolic pathway (Fig. 3D). Furthermore, the data suggests a strong remodelling of the TCA cycle towards a glyoxylate cycle. In this regard, amino acid breakdown and the propanoyl-CoA pathway are upregulated to replenish intermediates in the TCA/glyoxylate cycle. Finally, several enzymes of the glycolysis–gluconeogenesis axis were upregulated after NDUF-11 depletion. It is noticeable that both PYK-1, which is rate controlling in glycolysis, and PYC-1 and PCK-2*,* which are rate controlling in gluconeogenesis, were upregulated, suggesting a possible ‘futile metabolic cycle’. However, considering that our sample was from a pool of cells from the whole organism, it is reasonable to assume that different tissues might respond differently to reduced levels of NDUF-11. In this respect, we observed a downregulation of SDHA-2 (versus SDHA-1; Fig. 3D and Fig. S2) and ASB-1 (versus ASB-2; Fig. S2), which are germline-specific isoforms of complex II and the ATP synthase, respectively.

### Confirmation that NDUF-11 is a constituent of *C. elegans* mitochondrial complex I

**Fig. 4.**
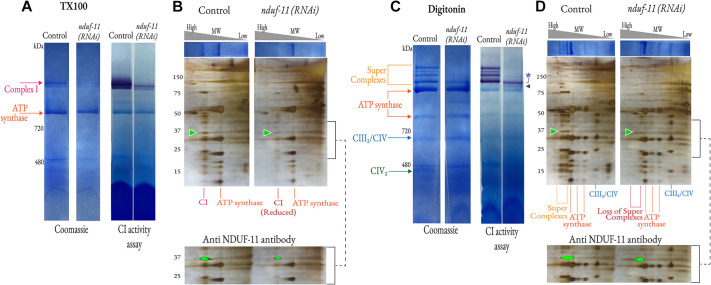
**Analysis of mitochondrial respiratory complexes.** (A) BN-PAGE analysis of TX-100 extracts of mitochondrial proteins. A total of 78 µg of solubilised mitochondria was loaded per lane and subjected to Coomassie staining and an in-gel CI activity assay. Top arrow, monomeric CI; bottom arrow, ATP synthase (CV). Gel lanes were cut vertically and reassembled manually before imaging; uncropped images are shown in Fig. S3. (B) 2D BN-PAGE analysis of TX-100 mitochondrial protein extracts. First dimension BN-PAGE Coomassie-stained gel lane [shown top; high to low molecular weight (MW) from left to right] was resolved on a denaturing LDS-PAGE gel and silver stained. Green arrowhead indicates the position of NDUF-11. Bracket indicates the region shown beneath, where antibody detection of NDUF-11 is superimposed on the silver-stained gel. The signal from the protein ladder in both images was used to align the superimposed result. Uncropped images are shown in Fig. S4. (C) BN-PAGE analysis of digitonin extracts of mitochondrial proteins. A total of 150 µg of solubilised mitochondria was loaded per lane and subjected to Coomassie staining and an in-gel CI activity assay. Positions of respiratory complexes were determined by in-gel activity assays (for CI, IV and V; Fig. S5A). The asterisk highlights the loss of higher molecular weight supercomplexes in the *nduf-11(RNAi)* group. The band indicated by a black arrowhead in the *ndufa-11(RNAi)* group probably represents the SCI_1_:CIII_2_ assembly. Gel lanes were cut vertically and reassembled manually before imaging; uncropped images are shown in Fig. S3. (D) 2D BN-PAGE analysis of digitonin mitochondrial protein extracts. First dimension BN-PAGE Coomassie-stained gel lane (shown top; high to low MW from left to right) was resolved on a denaturing LDS-PAGE gel and silver stained. Green arrowhead indicates the position of NUDF-11. Bracket indicates the region shown beneath, where antibody detection of NDUF-11 was superimposed on the silver-stained gel. The signal from the protein ladder in both images was used to align the superimposed result. Uncropped images are shown in Fig. S4. Data are representative of five independent experiments.

Experiments were conducted to confirm that the *C. elegans* NDUFA11 homologue is associated with complex I. Mitochondria from animals with native and depleted levels of NDUF-11 were analysed by BN-PAGE, using conditions optimised for detecting respiratory complexes and ATP synthase. As a first step, the membranes were subjected to harsh solubilisation conditions with the detergent Triton X-100 (TX-100) prior to non-denaturing BN-PAGE (Fig. 4A, left). The protein complexes were visualised in this first dimension using Coomassie staining (Fig. 4A, left), and complex I was visualised using an in-gel activity assay (Schägger, 2002) (Fig. 4A, right). In the former, the two most prominent bands were monomeric forms of complex I and the ATP synthase, which had been separated from their respective respiratory supercomplex and homodimeric forms by TX-100. Individual proteins of complexes resolved by BN-PAGE were separated in a second dimension by denaturating electrophoresis in lithium dodecyl sulfate (LDS-PAGE). The proteins were then visualised using silver staining and immunoblotting, identifying NDUF-11 as a component of the monomeric complex I (Fig. 4B). As predicted, NDUF-11 was reduced in samples obtained from *nduf-11(RNAi)*-treated mitochondria. This analysis also showed there was a corresponding lower recovery of intact and active complex I from extracts where NDUF-11 was depleted, while the ATP synthase was unaffected. The correlation between levels of NDUF-11 and complex I recovery suggest that the supernumerary subunit is required for stable complex assembly.

### Reduction of NDUF-11 leads to destabilisation of the respiratory supercomplexes

The integrity of the respiratory supercomplexes during BN-PAGE is partially dependent on the nature of the detergent used for solubilisation (Schägger, 2002). Thus, for these studies, TX-100 was replaced by the milder detergent digitonin, which is known to preserve the supercomplexes containing complex I (Jha et al., 2016; Suthammarak et al., 2010, 2009) (Fig. 4C). Equivalent proportions of extracts derived from mitochondria with wild-type and depleted levels of NDUF-11 were analysed using BN-PAGE. A series of high molecular weight respiratory supercomplexes were visualised using a combination of Coomassie Blue and in-gel complex I-activity staining of the native extract (Fig. 4C). Conversely, the NDUF-11-depleted samples contained only the lower molecular weight form of complex I-positive supercomplexes (Fig. 4C, asterisk) and, crucially, were devoid of higher molecular weight supercomplexes seen in the control.

Interestingly, NDUF-11-depleted samples showed a faint band below the supercomplexes which was not observed in the N2 control group (Fig. 4C, arrowhead). Importantly, the migration pattern of this band was significantly different from that of the monomeric form of complex I observed in samples extracted using TX-100 (Fig. 4C versus Fig. 4A and Fig. S3H). Although the apparent migration pattern of the monomers can differ depending on the type of detergent used, it should be noted that the worm mitochondrial digitonin extracts had negligible to nil levels of monomeric complex I, compared to levels in extractions of bovine mitochondria (Fig. S5B). Interestingly, the lower molecular weight complex I-positive supercomplex (i.e. the faint band highlighted by the arrowhead in Fig. 4) resembled that present in bovine mitochondria (Fig. S5B, white arrowhead), suggesting that it could be the SCI:III_2_ core unit. Similar observations have been previously reported in complex IV-deficient worms (Suthammarak et al., 2009). Notwithstanding this, both protein (Coomassie Blue) and complex I-activity staining showed that the total amount of complex I was reduced to levels consistent with the proteomic analysis (Fig. S2). The quantity and state of the ATP synthase were unaffected (Fig. 4C).

Second-dimension denaturing LDS-PAGE confirmed the presence of complex I subunits in the different bands assigned to high molecular weight respiratory supercomplexes (Fig. 4D). Immunoblotting confirmed the identity of NDUF-11 in all of these supercomplexes (Fig. 4D, lower panel). The resolution of the 2D blot was not sufficient to discern the presence or absence of NDUF-11 in the lower faint band (Fig. 4C, arrowhead) of NDUF-11-depleted samples.

Taken together, these results suggest that depletion of NDUF-11 destabilises respiratory supercomplexes, resulting in either their fragmentation within the membrane or their loss under mild extraction conditions. Thus, the data identifies a potential positive correlation between the amount of NDUF-11 with the levels and/or stabilities of complex I and its supercomplexes.

### Reduction of NDUF-11 affects mitochondrial morphology

**Fig. 5.**
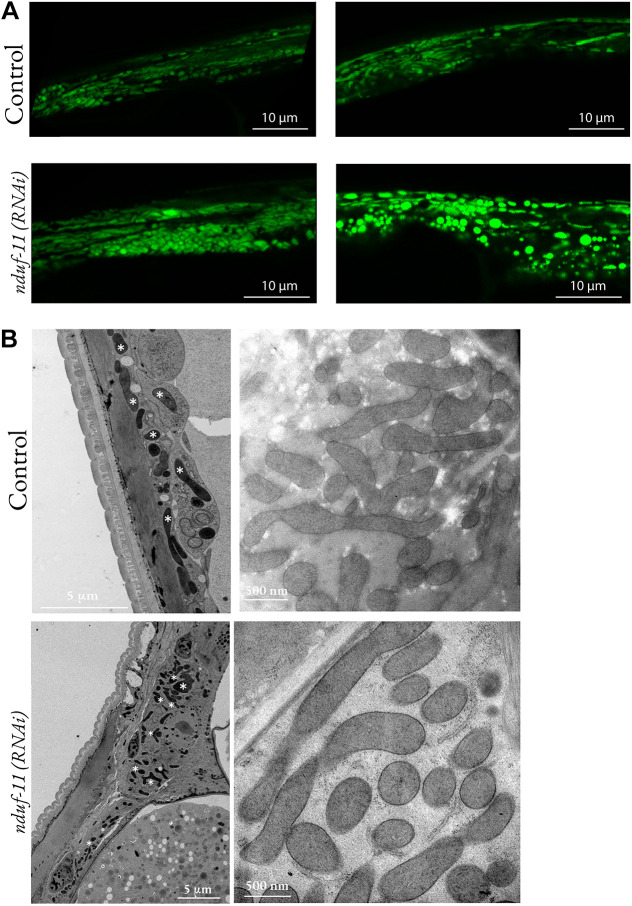
**Mitochondrial morphology of *nduf-11(RNAi)*-treated animals.** (A) Representative confocal images of GFP-labelled mitochondria in the body wall muscles (*Pmyo-3::mito::GFP*) of wild-type and *nduf-11(RNAi)* animals. A total of 20 worms were imaged in three independent experiments. (B) Electron micrographs of body wall mitochondria in wild-type and *nduf-11(RNAi)* adult animals. Images are representative of seven worms in each group from a single experiment. Some but not all mitochondria in the micrograph have been highlighted by white asterisks in the left panels.

A targeted GFP reporter was used to observe changes in mitochondrial morphology and distribution in body wall muscles. Compared to those of wild-type animals, NDUF-11-depleted mitochondria appeared fragmented and less reticulated (Fig. 5A); a possible indicator of a pathological condition. These morphological differences were also evident after inspection using transmission electron microscopy (TEM) of high-pressure frozen and freeze-substituted *C. elegans* worms (Fig. 5B). The analysis showed that NDUF-11-depleted mitochondria were more spherical compared to those in the control, which had a more elongated shape (Fig. 5B, right panels).

**Fig. 6.**
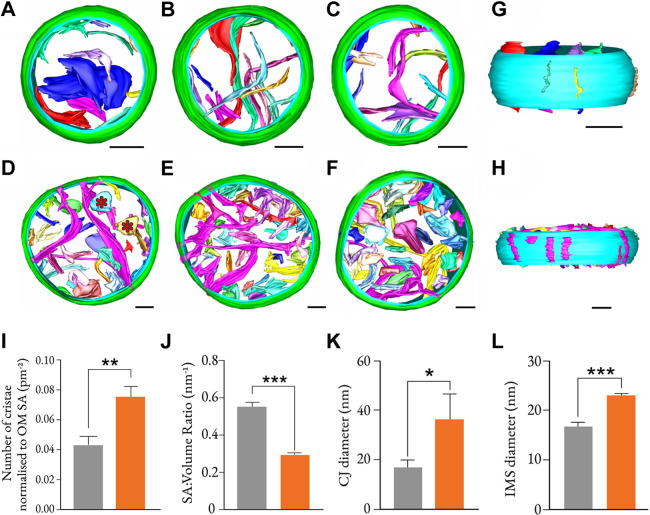
**NDUF-11 knockdown affects formation of lamellar cristae.** (A–F) Tomographic reconstruction and segmentation of representative wild-type (A–C) and *nduf-11(RNAi)* (D–F) mitochondria isolated from *C. elegans*. Wild-type mitochondria have lamellar cristae, whereas *nduf-11(RNAi)* mitochondria have more sac-like cristae, as indicated by the red asterisks in D. Green, outer membrane; cyan, inner membrane; each crista membrane has a different colour. Reconstructions were made using IMOD 4.9. Scale bars: 100 nm. See Movie 1 (control) and Movie 2 (RNAi). (G,H) Side view of a wild-type (G) and *nduf-11(RNAi)* (H) mitochondrion from A and E, respectively, with outer membrane hidden, revealing slit-like CJs characteristic of *C. elegans* mitochondria. Junctions in *nduf-11(RNAi)* mitochondria are visibly wider than those in the wild-type mitochondria. Scale bars: 100 nm. (I) Number of cristae per mitochondrion (normalised to outer membrane surface area, OM SA) from wild-type and *nduf-11(RNAi)* mitochondrial reconstructions shown in panels A–F (*n*=3 mitochondria for each condition). (J) Crista surface area (SA):volume ratio. Mesh SA and volume inside the mesh of each membrane from mitochondrial reconstructions shown in A–F were calculated computationally (*n*=3 mitochondria for each condition; a total of *n*=37 wild-type and *n*=105 RNAi-treated crista were analysed). (K) Diameter of CJs. CJs were measured from the same data used to generate the images in A–F (*n*=3 mitochondria for each condition). (L) IMS diameter. IMS was measured from the same data used to generate the images in A–F (*n*=3 mitochondria for each condition, with four points per mitochondrion analysed). Data in I–L are shown as mean±s.d., with wild type shown in grey and *nduf-11(RNAi)* shown in orange. *P≤0.05, **P≤0.01, ***P≤0.001 (unpaired, parametric Student's *t*-test).

In order to explore the mitochondrial interior and ultrastructure in greater detail, samples of isolated mitochondria were vitrified and subjected to cryo-ET. The tomographic reconstructions (Fig. 6A–C,G; Movie 1 for controls; Fig. 6D–F,H; Movie 2 for RNAi treated) show that mitochondria with depleted NDUF-11 (with reduced and/or destabilised levels of complex I and its supercomplexes) contain a higher number of identifiable cristae compared to control specimens (Fig. 6I). Interestingly, the frequency of cristae junctions (CJs) in the NDUF-11-depleted mitochondria was found to be similar to that of the control (Fig. S6A), suggesting that a high number of cristae are fragmented and not connected to the IMM. This can be seen directly in the tomographic images (Fig. 6D–F, marked with asterisks in D), where cristae from NDUF-11-depleted mitochondria have lost the classical mitochondrial lamellar morphology, resulting in disconnected sac-like crista with a lower surface area to volume ratio (Fig. 6J). Consequently, the total cristae surface area and volume are increased (Fig. S6B,C), despite the unchanged average dimensions (surface area or volume) of them individually (Fig. S6D,E).

Finally, perturbation of complex I and its supercomplexes correlated with a widening of the CJs (Fig. 6G,H,K; Fig. S6F), and an increased separation between the outer and inner membranes (i.e. widening of the IMS; Fig. 6L). Since the MICOS complex, located at CJs, is known to mediate cristae and IMM remodelling (Stephan et al., 2020), we revisited the mass spectrometry dataset (Fig. 3A–C) to assess the expression levels of key components of this complex (Head et al., 2011), namely IMMT-1, IMMT-2, MOMA-1, CHCH-3, F54A3.5 and W04C9.2. Only IMMT-2, a homologue of yeast Mic60, was significantly downregulated after RNAi treatment (−2.15 log_2_ fold change, *P*=0.003, in the mitochondrial fraction). However, it should be noted that the *C. elegans* Opa1 homologue EAT-3 remained unchanged after *nduf-11* silencing. Overall, our data show that NDUF-11 and its loss induce profound changes in the internal membrane architecture of mitochondria, possibly as a consequence of metabolic remodelling.

### Maintenance of complex I and its supercomplexes is required for optimised mitochondrial respiration and mitigation against excessive reactive oxygen species production

**Fig. 7.**
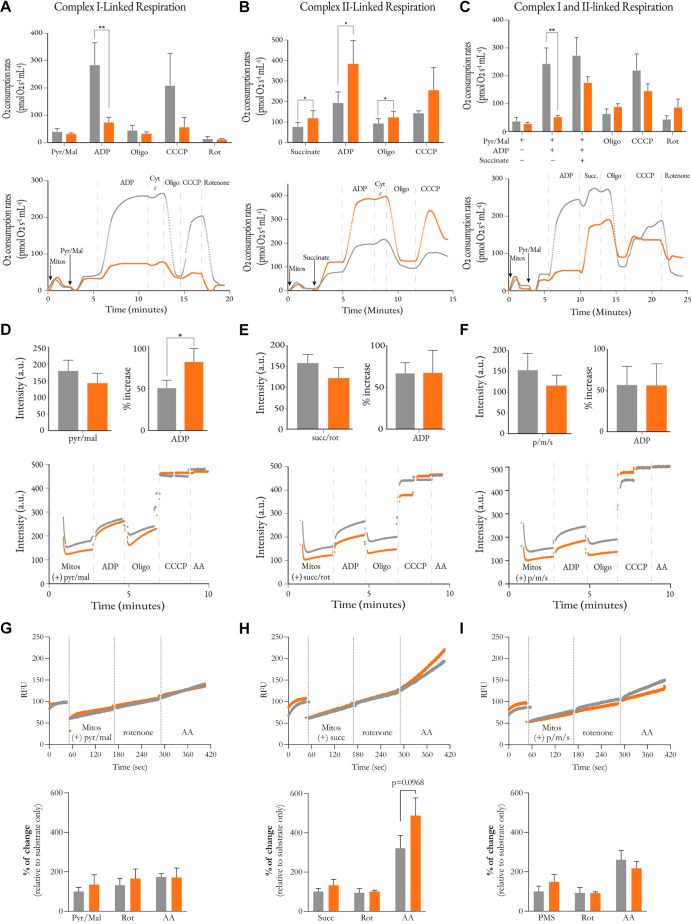
**Bioenergetic analysis of mitochondria after *nduf-11(RNAi)* treatment*.*** (A–C) Assessment of mitochondrial respiration by monitoring oxygen consumption in isolated fractions. Wild type is shown in grey and *nduf-11(RNAi)* is shown in orange. Mitochondria were energised with either (A) complex I-linked substrates (pyruvate and malate, Pyr/Mal), (B) complex II-linked substrates (succinate) or (C) both. ADP, inhibitors and uncoupler were sequentially added to assess mitochondria fitness (dashed lines in the lower panels). Line plots show mean values of 3–5 independent experiments. Bar charts show mean±s.e.m. of four independent experiments. Differences between groups for each respiratory state were assessed using a paired Student's *t*-test. (D–F) Assessment of mitochondrial respiration by monitoring membrane potential using TMRM in isolated fractions. Wild type is shown in grey and *nduf-11(RNAi)* is shown in orange. Mitochondria were energised as in A–C. Line plots show mean values of TMRM signal from 3–5 independent experiments. Bar charts shown as mean±s.e.m. of four independent experiments. Differences between groups for each respiratory state were assessed using a paired Student's *t*-test. (G–I) Production of hydrogen peroxide by isolated mitochondria, monitored using Amplex Red. Wild type is shown in grey and *nduf-11(RNAi)* is shown in orange. Mitochondria were energised as in A–C. Line plots show traces from a single experiment, representative of four (G,H) or three (I) repeats. Bar charts represent relative fluorescent values at specific timepoints (see Materials and Methods) and are shown as mean±s.e.m. of four independent experiments. Differences between groups in the presence of each inhibitor were assessed using a paired Student's *t*-test. *P<0.05; **P<0.01. AA, antimycin A; a.u., arbitrary units; Cyt c, cytochrome *c*; Mitos, mitochondria; Oligo, oligomycin; p/m/s, pyruvate, malate and succinate; RFU, relative fluorescence units; Rot, rotenone; Succ, succinate.

Next, we explored the consequences of NDUF-11 depletion, and subsequent respiratory complex destabilisation, on bioenergetic fitness. Thus, the respiratory performance of isolated mitochondria was assessed based on their oxygen consumption and membrane potential. Upon addition of the exogenous complex I substrates pyruvate and malate, similar rates of basal respiration were measured for mitochondria with native or depleted levels of NDUF-11 (Fig. 7A, top panel). When stimulated by the addition of ADP, the rate of respiration increased to drive OXPHOS. This activation dropped from 6.9-fold to a modest 2.3-fold in mitochondria with reduced levels of NDUF-11. In both cases, the addition of exogenous cytochrome *c* during ADP-sustained respiration (Fig. 7A, bottom panel) elicited little change, indicating that the outer membrane was largely intact and had retained endogenous cytochrome *c*. Therefore, the lowered respiratory capacity was not a consequence of mitochondria outer membrane permeabilisation. In contrast to mitochondria with wild-type levels of NDUF-11, those depleted of NDUF-11 also failed to respond to the uncoupler CCCP after addition of oligomycin. This suggests that the impairment was associated with the ETC and not the phosphorylation system.

In a separate experiment, to confirm that the effects described above were specific to complex I, its activity was inhibited using rotenone, and mitochondria were energised with the complex II substrate succinate (Fig. 7B). Under these conditions, NDUF-11-depleted mitochondria responded to the addition of both ADP and CCCP – achieved by bypassing the compromised complex I. Surprisingly, the complex II rescued respiration rates were consistently higher when compared to those of the control (Fig. 7B). In this case, the respiratory control ratio (RCR; ADP-stimulated over oligomycin-inhibited respiration), an indicator of the coupling efficiency between respiration and ADP phosphorylation, was significantly higher when NDUF-11 was depleted (3.06±0.12 versus 2.07±0.15; mean±s.e.m.; *P*=0.002).

**Fig. 8.**
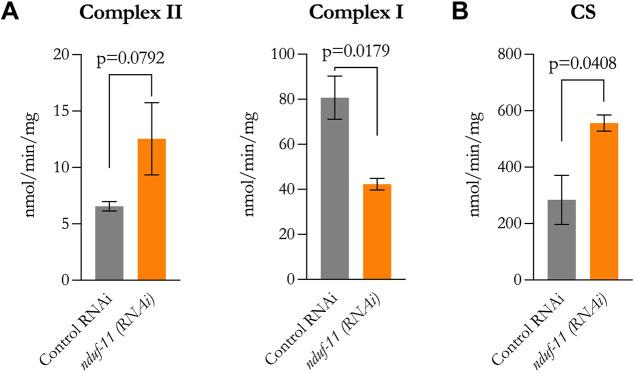
**Enzymatic activities for complex I, complex II and citrate synthase in isolated mitochondrial fractions.** For details of the assays, see the Materials and Methods section. (A) Mean±s.e.m. of three independent experiments for complex II and complex I activities. (B) Mean±s.e.m. of three independent experiments for citrate synthase activity. Differences between groups were assessed using a Student's *t*-test, and the resulting *P*-values are shown in the figure.

We next measured the individual enzymatic activities of complex I and II in isolated mitochondrial fractions from N2 control and *nduf-11(RNAi)*-treated animals (Fig. 8). In line with the proteomic data, we observed a marginal increase in complex II and a significant decrease in complex I activities (Fig. 8A). This also confirmed the respiration data, where the reduced RCR seen with pyruvate and malate in the NDUF-11-depleted mitochondria reflects the low activity of complex I. While this is sufficient to maintain rates of basal respiration, where the PMF restricts electron flow, this is not the case in the presence of ADP, which draws from the PMF and places a high demand on complex I; in this case the required high rates of complex I activity cannot be achieved when NDUF-11 is depleted. Conversely, with succinate as substrate, the increased activity of complex II has little effect on the basal rate of respiration but increases ADP-dependent respiration leading to an increase in RCR.

Usually, mitochondrial biochemical activities are normalised to citrate synthase (CS) activity; however, we observed a significant increase of CS activity in NDUF-11-depleted mitochondria (Fig. 8B), precluding its use for normalisation. Nonetheless, this increase in CS activity is in agreement with the upregulation detected in the proteomic analysis (2.44-fold change, *P*=0.036; Fig. 3C,D, cts-1).

The data show that mitochondria with depleted NDUF-11 levels are respiration competent (high RCR for complex II-linked respiration) and suggest that increased succinate levels could rescue mitochondrial respiration *in vivo*. To test this notion, mitochondria were incubated in pyruvate and malate with ADP prior to the addition of succinate in the absence of rotenone to better represent those conditions experienced by mitochondria *in vivo* (Fig. 7C). Succinate addition produced only a minor rate increase in unaffected mitochondria but was able to restore ADP-sustained respiration rates in NDUF-11-depleted, complex I-compromised mitochondria, albeit not to wild-type values. Therefore, it can be speculated that tissues able to modulate their metabolism towards succinate production, as globally shown by our proteomic data (Fig. 3; Fig. S2), are better able to compensate for the loss of complex I and supercomplexes upon *nduf-11* silencing.

To continue, the transmembrane membrane potential of the inner membrane (Δσ) was investigated using the fluorescent potentiometric probe TMRM (Fig. 7D–F). The maximum observed levels of Δσ were approximately the same, irrespective of levels of NDUF-11 or choice of the respiratory substrate (complex I- or complex II-linked). However, depolarisation by the addition of ADP was greater in NDUF-11-depleted mitochondria compared to that in the control when mitochondria were energised with complex I-linked substrates (Fig. 7D). Δσ_ADP_ reflects the equilibrium attained between PMF utilisation by the ATP production machinery (proton influx) and its generation by the ETC (proton pumping). Therefore, the reduced ability of mitochondria depleted of NDUF-11 to respire through complex I leads to a greater reduction in Δσ upon ADP addition because the ETC pumps fewer protons by comparison to those of the wild-type. This is in contrast to complex II-linked respiration, which in this respect was unaffected (Fig. 7E). When pyruvate, malate and succinate were simultaneously added under ‘combined’ respiration conditions (Fig. 7F), ADP depolarisation was also unaffected. The data confirm that bypassing complex I through the addition of succinate rescues the reduced bioenergetic fitness attributed to compromised complex I and respiratory supercomplex maintenance due to limited levels of NDUF-11.

Finally, we measured hydrogen peroxide production using Amplex Red to determine whether the impaired respiration displayed by the compromised mitochondria led to an elevation in reactive oxygen species (ROS) by the ETC. When mitochondria were energised through complex I by pyruvate and malate, hydrogen peroxide production was unaffected even in the presence of the ETC inhibitors rotenone (inhibitor of complex I) and antimycin A (inhibitor of complex III) (Fig. 7G). By contrast, when energy was supplied via complex II with succinate, the mitochondria depleted of NDUF-11 experienced a marginal, albeit not statistically significant, increase of hydrogen peroxide production after antimycin A addition, likely caused by the increased flow of electrons through the ETC (Fig. 7H). When NDUF-II-depleted mitochondria were energised with pyruvate, malate and succinate simultaneously (‘combined’ respiration; Fig. 7I), the hydrogen peroxide production was unaffected or decreased to some degree when compared to that in the control group.

## DISCUSSION

NDUFA11 is one of the many supernumerary subunits of mammalian respiratory complex I, with homologues in *Neurospora crassa* (NUO21.3b) (Hirst et al., 2003) and plants (B14.7, also known as OEP163 in *Arabidopsis*) (Wang et al., 2012). Interestingly, NDUFA11 also shares homology with the essential Tim17 family (Žárský and Doležal, 2016), including Tim17 and Tim23, which are core subunits of the TIM23 complex, and Tim22 at the centre of the TIM22 complex (reviewed by Needs et al., 2021). The importance of NDUFA11 as a complex I subunit was established in *N. crassa* and human cell culture studies, which revealed that impaired NDUFA11 levels lead to the incomplete assembly of complex I (Andrews et al., 2013; Nehls et al., 1992). It has been shown that NDUFA11 acts as an assembly factor to help in the incorporation of the distal component modules ND-4 and ND-5 to the membrane arm before the final addition of the catalytic N-module to the matrix-facing side (Andrews et al., 2013). Our experimental design can only identify competent complex I and derived structures of the mature complex with catalytic activity, but is not able to distinguish sub-assembly stages. Therefore, our data suggest that the residual NDUF-11 upon RNAi treatment is present in the observed (mature) complex I and, consequently, the total levels of complex I are limited by the supply of NDUF-11.

We identified an NDUFA11 homologue in the multicellular model organism *C. elegans* (NDUF-11) and targeted this protein to destabilise complex I, as well as to interfere with its ability to form respiratory supercomplexes. This in turn enabled an exploration of the consequences of this disruption from a biochemical perspective through to whole organism physiology. The results highlight the importance of ETC organisation for respiratory fitness, mitochondrial ultra-structure and function, and ultimately healthy development.

Perhaps unsurprisingly, deletion of *nduf-11* caused a developmental arrest, and its depletion disrupted complex I activity with a reduction in complex I levels and a corresponding complete loss in the recovery of respiratory supercomplexes. These results are consistent with previous work indicating that the integrity of complex I and the respiratory supercomplexes are interdependent (Jang and Javadov, 2018). They are also in agreement with NDUFA11 being an intrinsic assembly and stabilisation factor for complex I and the respiratory supercomplexes (Andrews et al., 2013; Guerrero-Castillo et al., 2017). They also support studies elsewhere suggesting that loss of the interface between complex I and complex III renders the complex immature and prone to degradation (Acin-Pérez et al., 2004; Protasoni et al., 2020).

The milder phenotype displayed by worms with a reduction, but not elimination, of NDUF-11 enabled us to produce sufficient biomass for biochemical studies. This, in turn, allowed us to investigate how complex I, when compromised in structure and supercomplex formation, affects physiology and health in a multicellular organism. Despite the major loss of complex I and its ability to organise into supercomplexes, the bioenergetic performance baseline of the affected mitochondria remained largely unchanged. The effects were only revealed when mitochondria were challenged by an increase in energy demand. This is particularly easy to assess *in vitro* in isolated mitochondrial fractions by incubation with exogenous ADP or, alternatively, by addition of uncouplers, which also leads to mitochondrial depolarisation. Overall, the observed limitation in respiratory activity could explain why worms displayed a developmental arrest phenotype, as discussed below.

Succinate was able to bypass and rescue (via complex II) the complex I-specific deficiencies, indicating that other respiratory complex assemblies must persist more or less intact, consistent with the results of our BN-PAGE and proteomic analyses. However, when succinate was added exogenously to mitochondria isolated from *nduf-11(RNAi)* animals, a drastic increase in respiration was observed, indicating that significant remodelling had occurred. The observed increase in succinate-driven respiration in mitochondria from *nduf-11(RNAi)* animals seems to be explained by one, two or all of the following: (1) an increased succinate entry into the mitochondria through the dicarboxylic carrier (SLC-25A10), whose expression was increased by 104%; (2) the upregulation of ubiquinone/cytochrome *c* (CYC-2.1; increased 110%); and (3) the 25–75% increase in expression of complex II subunits.

Interestingly, succinate did not fully rescue the ADP-sustained respiration when added after the complex I-linked substrates pyruvate and malate. Under these experimental conditions, the residual levels of complex I in NDUF-11-depleted mitochondria are insufficient to support maximal rates of respiration, and a greater contribution from succinate is expected. However, the residual complex I activity will lead to the production of some oxaloacetate, which is an extremely potent inhibitor of complex II (Stepanova et al., 2016; Zeyelmaker and Slater, 1967). This will reduce rates of succinate oxidation relative to native levels and so lower the maximal respiration, as observed. Although the same inhibitory effect of oxaloacetate on complex II activity would be experienced by wild-type mitochondria, the higher levels of complex I in this group can fully sustain maximal rates of respiration, and therefore the contribution required from complex II would be minimal. A further presumable consequence of lower complex I activity is the reduction in levels of ATP, because succinate alone cannot drive respiration *in vivo* where it is made via the citric acid and/or glyoxylate cycles, whose operation generates NADH and, thus, requires complex I. Therefore, reduction in levels of ATP could also be contributing to the observed phenotype.

The changes observed in isolated mitochondria reflect profound physiological adaptations of the whole worm organism upon NDUF-11 depletion with a corresponding reduction of complex I and loss of its supercomplexes. Our proteomic analyses indicate that affected animals also shut down their fatty acid biosynthesis and remodel the TCA cycle towards the glyoxylate shunt. The latter is a characteristic of worm metabolism in the quiescent dauer state, an alternative developmental stage that promotes survival under stress, for example when food is scarce (Braeckman et al., 2009), as well as in L1 stage, prior to a metabolic shift towards aerobic respiration required for entry into L2 and later developmental stages (Braeckman et al., 2009). The purpose of the glyoxylate cycle is to increase nutrient stores via acetyl-CoA conversion towards succinate through the use of fatty acids or acetate as carbon sources, which can thereafter be used in anaerobic respiration. The co-existence of impaired aerobic respiration with a shift towards anaerobic processes not only explains the downregulation of fatty acid biosynthesis, but also why the NDUF-11-depleted animals are able to reach adulthood, albeit at slower rates.

So why do the first generation of worms with compromised complex I organisation progress through the L2 stage instead of entering the dauer stage? In addition to favourable environmental cues (abundant food source), affected animals have reduced levels of DAF-18, which has previously been shown to be associated with defective dauer entry, extended lifespan and suppressed fat accumulation (Gottlieb and Ruvkun, 1994). Moreover, *daf-18* negatively regulates germline insulin/IGF-1 signalling during optimal nutrient uptake (Narbonne et al., 2015). Therefore, it seems plausible that the reduced mitochondrial fitness and respiratory capacity, caused by depleted NDUF-11, is sufficient for development into adulthood – albeit with reduced body size and low progeny numbers. The progeny were, however, sterile, most likely because the bioenergetic demands for oocyte maturation (Charmpilas and Tavernarakis, 2020) and sperm motility and function (Yen et al., 2020) are such that full mitochondrial capacity is required.

Similarly, developmental arrest in the *nduf-11* knockout can be attributed to poor mitochondrial fitness resulting from loss of complex I and its supercomplexes. Maturation to the L3 and L4 stages is associated with increased energy demand, consistent with the metabolic shift from the glyoxylate shunt towards aerobic metabolism after transit through the L2 stage (Tsang and Lemire, 2003). Moreover, knockdown of ETC subunits results in development arrest at multiple stages (Lee et al., 2003; Tsang et al., 2001). Additionally, ubiquinone deficiency leads to L2-stage arrest (Jonassen et al., 2001), consistent with the importance of complex I (and its assembled states) during worm development.

Another remarkable consequence of *nduf-11(RNAi)* treatment is the remodelling of the inner mitochondrial morphology. Both the qualitative and quantitative tomography data clearly demonstrate that cristae suffer from a loss of their native lamellar morphology, which is likely to have detrimental effects on mitochondrial health as crista shape has been found to dictate respiratory efficiency. Significantly, aberrations in crista morphologies are associated with numerous devastating diseases including neurodegeneration and cancer (Ogando et al., 2019; Wu et al., 2019).

The perturbation of crista morphology could potentially result from reduced production of MICOS or ATP synthase components. MICOS complexes (located at CJs) mediate crista formation (Stephan et al., 2020), and arrangement of ATP synthase dimers into rows is instrumental in formation of sharp, curved ridges characteristic of lamellar cristae (Davies et al., 2012). However, mass spectrometry and BN-PAGE showed that the latter was unaffected. In contrast, although most of the MICOS subunit levels remained constant, the subunit responsible for marking the nascent sites of CJ formation and contact sites between the inner and outer mitochondrial membrane (Stephan et al., 2020), the Mic60 homologue IMMT-2, was significantly downregulated after reduction of NDUF-11. Interestingly, *nduf-11* loss corresponded to widening of the CJs. Despite this, there was no evident cytochrome *c* release, which explains why the bioenergetic fitness was maintained.

In conclusion, our work shows that destabilisation of complex I and the concomitant destabilisation and loss of respiratory supercomplex formation has a profound effect on bioenergetic capacity, mitochondrial metabolism and morphology, which severely impairs higher-level physiological functions. Affected worms are unable to meet the energy requirements associated with gonad differentiation and healthy development into adulthood.

The complete ablation of NDUF-11 reveals its essential role in organismal health and proliferation, which we attribute to a catastrophic loss of complex I levels. The milder effects of depletion, which supported *C. elegans* viability to the adult stage, allowed us to examine the consequences of complex I destabilisation and loss of respiratory supercomplexes. The prognosis for health, while not lethal, remains severe – as we have shown. We speculate that the extent of complex I destabilisation and loss of supercomplexes would have a corresponding effect on the severity of human mitochondrial disease. Indeed, patients with genetic abnormalities associated with *NDUFA11* present with encephalocardiomyopathy and fatal infantile lactic acidemia (Berger et al., 2008). The effects of perturbed *ndufa-11* expression described here provide a molecular basis for these, and potentially other afflictions involving supercomplex breakdown. Perhaps there are also indirect consequences of complex I supercomplex perturbation to be considered. We show that the respiratory rebalancing for restorative OXPHOS, by increased expression and activity of complex II, comes with the potential for greater ROS production. It may well be that the side-effects of this physiological ‘cure’ for problems in complex I and supercomplex stability may themselves be problematic, and thus responsible for deterioration of health.

## MATERIALS AND METHODS

### Strains and nematode culture

The *C. elegans* strain N2 Bristol was used as the wild-type strain. Additional strains were obtained from the Caenorhabditis Genetics Center (CGC; University of Minnesota, MN, USA): SJ4103 (*zcIs14 [myo-3::GFP(mit)]*); CGC43 (*unc-4(e120)/mnC1[dpy-10(e128) unc-52(e444) umnIs32] II*). The CRISPR-Cas9 generated *cr51* knockout allele of *B0491.5* was balanced *in trans* with *umnIs32*. *Escherichia coli* strains OP50 and NA22 were used for worm propagation, and *E. coli* strain HT115 was used for feeding RNAi.

*C. elegans* stocks were maintained at 20°C on NGM agar plates using standard methods (Brenner, 1974). Bulk liquid preparations of *C. elegans* were fed a suspension of NA22 bacteria in S-basal complete medium (Stiernagle, 2006; Sulston and Hodgkin, 1988) and incubated at 20°C in a shaker incubator at 200 r.p.m. Synchronous worm populations were obtained by collecting eggs from adult hermaphrodites after alkaline hypochlorite treatment and allowing them to hatch overnight into L1 larvae in M9 buffer (3.0 g/l KH_2_PO_4_, 6.0 g/l Na_2_HPO_4_, 0.5 g/l NaCl, 1.0 g/l NH_4_Cl) lacking food (Sulston and Hodgkin, 1988).

All experiments were carried out in adult worms, except the collection of data shown in Table S1, which represents worms in different developmental stages after second-generation developmental arrest.

### Homology modelling

Modeller 9.12 (Sali and Blundell, 1993) was used to obtain a homology model of B0491.5 based on the NDUFA11 protein structure found in respirasome structure PDB:5GUP (Wu et al., 2016). A total of 5000 individual models were constructed and scored according to their Discrete Optimised Protein Energy (DOPE) value. The top 1% of models were retained and analysed using the Gromacs (Berendsen et al., 1995) tool gmx cluster to identify clusters of well-scoring structures. The model with the best score from the largest cluster was selected.

### Molecular dynamics simulations

The stability of the homology model was validated using atomistic molecular dynamics simulation. The protein was described using the CHARMM36 force field (Best et al., 2012) and embedded into a POPC membrane and explicit solvent using CHARMM-GUI (Jo et al., 2007; Lee et al., 2016). The system was energy minimised using the steepest descents method, then equilibrated with positional restraints on heavy atoms for 100 ps in the NPT ensemble at 310K with the V-rescale thermostat and semi-isotropic Parrinello–Rahman pressure coupling (Bussi et al., 2007; Parrinello and Rahman, 1981). A production simulation was run using 2 fs time steps over 200 ns, using GROMACS 2018 (Berendsen et al., 1995). Root-mean-square deviation (RMSD) and root-mean-square fluctuation (RMSF) analyses were run using the Gromacs tools gmx rms and gmx rmsf. All molecular models were represented using PyMOL (pymol.org).

### CRISPR-Cas9

The *cr51* knockout allele of *B0491.5* was obtained using CRISPR-Cas9 following the *dpy-10* co-conversion protocol developed by the Fire lab (Arribere et al., 2014). Targeted deletion of *B0491.5* was achieved using a pair of guide RNA (gRNA) clones pPK874 and pPK875. These were obtained by ligating complementary primer pairs (PK1801/PK1802 and PK1803/PK180; Table S3) with asymmetric BsaI overhangs into the BsaI site of pRB1017 (Addgene: 59936, deposited by Andrew Fire). The single-strand PK1805 oligodeoxynucleotide (ssODN; Table S3) repair template shares sequence homology extending beyond both the selected *B0491.5* PAM sites and was designed to replace the deleted region with a seven-nucleotide sequence to terminate any potential translational readthrough (Fig. S7). DNA sequencing (MWG Eurofins) confirmed the presence of a 146 bp deletion disrupting exons 1 and 2 and insertion of the 7 bp sequence.

### RNA interference

RNA interference (RNAi) was performed by using clones obtained from an RNAi feeding library (Kamath and Ahringer, 2003; Source Bioscience, Nottingham, UK) and an empty vector control. To perform RNAi to deplete *nduf-11*, synchronised N2 L1 larvae were grown on *B0491.5(RNAi)* or empty-vector NGM feeding plates. Individual animals were plated onto fresh RNAi plates when they reached the L4 stage. Adult RNAi-treated worms were then transferred every 12 h to fresh RNAi plates until egg-laying ceased. Progeny were examined and counted 24 h after the transfer of adults to fresh plates.

To obtain sufficient quantities of RNAi-treated worms for biochemical analyses, four 1 l cultures of 2×TY (16 g/l tryptone, 10 g/l yeast extract and 5.0 g/l NaCl) were each inoculated with 50 ml of a saturated culture of *B0491.5* RNAi feeding bacteria and induced with 0.4 mM IPTG after reaching OD_600_=0.4. Induced cultures were grown for an additional 2 h before being harvested and resuspended in half of their original volume in S-basal complete medium supplemented with 100 μg/ml ampicillin and 0.4 mM IPTG. Cultures were inoculated with a synchronous population of L1 larvae (∼4×10^6^ worms) and grown in a shaker incubator at 200 r.p.m. at 20°C for 4 days or until they reached the adult stage.

### Lifespan assays

Lifespan assays were performed in the presence of 5-fluorodeoxyuridine (FUdR), as described previously (Lithgow et al., 1995). L4-stage larvae were placed on FUdR plates seeded with RNAi bacteria and incubated at 20°C. In survival curves, day 1 represents the first day of adulthood. Animal viability was scored every other day by tactile stimulation. Animals that were lost or ruptured were not included in analyses (Chen et al., 2007). Survival data was analysed using a Kaplan–Meier plot.

### Mitochondrial isolation

Worms were harvested from liquid culture and separated from debris by sucrose floatation (Sulston and Hodgkin, 1988); ∼2–6 ml packed worm volume per 0.5 l of culture was obtained. Worms were washed extensively in M9 buffer (3.0 g/l KH_2_PO_4_, 6.0 g/l Na_2_HPO_4_, 0.5 g/l NaCl and 1.0 g/l NH_4_Cl) to restore osmolarity before being resuspended in 10 ml collagenase buffer (100 mM Tris-HCl pH 7.4 and 1 mM CaCl_2_) with 1 U/ml collagenase (Sigma, C0773) and incubated for 2 h at 20°C with gentle agitation. After incubation, worms were harvested by centrifugation at 160 ***g*** and washed in STEG/M buffer (220 mM mannitol, 70 mM sucrose, 5 mM Tris-HCl pH 7.4 and 1 mM EGTA) before being resuspended in 10 ml of STEG/M(+) [STEG/M supplemented with 1 mM PMSF in methanol and 1% fatty acid-free BSA (Sigma, A6003)]. Worms were homogenised in a 50 ml fitted glass/Teflon power-driven Potter–Elvejhem homogeniser before being centrifuged at 750 ***g*** for 15 min at 4°C. The supernatant was transferred to a fresh tube, and the pellet was re-extracted before pooling supernatants and collecting mitochondria by centrifugation at 12,000 ***g*** for 15 min at 4°C. Mitochondria were resuspended and washed in 30 ml STEG/M buffer before centrifugation at 750 ***g*** for 15 min, 4°C. The supernatant was centrifuged at 12,000 ***g*** for 15 min, 4°C and the final pellet containing mitochondria was resuspended in 150 μl STEG/M buffer. Protein concentration was determined using a BCA protein kit (Thermo Fisher Scientific). Mitochondria were flash frozen in liquid N_2_ before storage at −80°C or used no later than 4–6 h post-isolation for physiological assays.

When collecting samples for mass spectrometry, worms were processed as described above, and samples were harvested at specific steps during the protocol. For the cytosolic fraction, a sample was collected from the supernatant after the first high-speed spin, therefore not including nuclear or membrane proteins. For the mitochondrial fraction, a sample was collected from the pellet of the first high-speed spin after performing a single wash.

### Confocal fluorescence microscopy

Worms were placed in a 5 μl drop of 1 mM levamisole on a 4% agarose pad. After 30 min, a coverslip was placed over the worms. Images were collected using a Leica TCS SP8 AOBS confocal laser scanning microscope. Images were analysed using ImageJ software (Bethesda, MD, USA).

### Transmission electron microscopy

Worms were immersed in NA22 *E. coli* medium in a 0.2 mm deep gold-coated copper carrier prior to high-pressure freezing with a Leica EMpact2 high-pressure freezer (Leica Microsystems, Vienna, Austria) (Verkade, 2008). Worms were freeze substituted for 2 h at −90°C with 1% osmium tetroxide and 0.1% uranyl acetate in acetone using a Leica EM AFS2 freeze substitution processor (Leica Microsystems). After a temperature ramp to 0°C, specimens were washed with a graded series of acetone/Epon resin, followed twice by Epon resin alone (Verkade, 2008), and were polymerised at 60°C between two Aclar (polychlorotrifluoroethylene) sheets (Aclar UltRx 2000; EMS Diasum 50426) to flatten worms and facilitate longitudinal sectioning. 1 μm thick sections were obtained and stained with Methylene Blue prior to obtaining ultrathin 70 nm sections using a Reichert Ultracut S ultramicrotome (Leica Microsystems). Sections were stained with lead and uranyl salts, and images obtained using a Tecnai T12 microscope (Thermo Fisher Scientific).

### Cryo-ET of isolated mitochondria

Holey carbon EM grids (Quantifoil, Jena, Germany) were glow-discharged under vacuum using an ELMO TM Glow Discharge System (Cordouan Technologies, France) according to the manufacturer's instructions. The isolated mitochondrial suspension (5–10 mg/ml) was mixed 1:1 with a stock solution of 10 nm protein A–gold fiducial markers (Aurion, Wageningen, The Netherlands), and 3 µl was immediately applied to a glow-discharged grid. Grids were blotted for 1–1.5 s, followed by plunge-freezing in liquid ethane using a Vitrobot Mark IV (Thermo Fisher Scientific). Samples were placed in a grid storage box and stored under liquid nitrogen prior to viewing.

Cryo-ET was performed using a FEITM TalosTM, equipped with a 200 kV FEG (Thermo Fisher Scientific), K2 DED and GIF BioQuantum LS energy filter operated with a slit width of 20 eV (Gatan, Pleasanton, USA). Dose-fractionated tomograms (2–5 frames per tilt) were collected at a magnification of 49k× (corresponding to a pixel size of 2.8 Å), typically collected from +60° to −60° with tilt steps of two degrees. The total dose per tomogram was <120 e^−^/Å^2^. Gold fiducial markers were used to align tomograms, and volumes were reconstructed using the IMOD software (Kremer et al., 1996). Tomogram contrast was enhanced by non-linear anisotropic diffusion (NAD) filtering in IMOD.

### Mitochondrial segmentation and morphometric analysis

Mitochondrial membranes were segmented in IMOD, through drawing and interpolation of closed contours. Using the imodmesh algorithm, contours were meshed at a *z*-increment of four, with a point reduction tolerance of two.

The mesh surface area of each membrane was computed by adding the areas of all triangles in the mesh; the volume inside the mesh of each membrane was computed by adding the volumes of tetrahedra formed between each mesh triangle and a central point within a capped mesh. Surface areas and volumes were normalised to that of the outer membrane (OM), before calculation of surface area:volume ratios. For display purposes, point reduction tolerance and *z*-increment were modified for the inner and outer membrane to give a smooth appearance, but these values were not used in calculations.

The diameter of the IMS (at four locations) and each segmented in-plane crista junction (CJ) was measured manually at the top, bottom and middle of each mitochondrion/junction, and an average was calculated. The number of CJs in each model file was normalised to the OM surface area by calculating the frequency per nm². Mean crista SA and volume, IMS width and CJ diameter for Fig. 6 and Fig. S3 were calculated from *N*=3 mitochondria for each condition, with *n*=37 (wild-type) and *n*=105 (NDUF-11 depleted) cristae analysed; *N*=3 mitochondria for each condition, *n*=4 points per mitochondrion analysed; and *N*=3 mitochondria for each condition, with *n*=37 (wild-type) and *n*=55 (NDUF-11 depleted) CJs analysed, respectively. All data for wild-type and *nduf-11(RNAi)* tomograms were compared using unpaired, parametric Student's *t*-tests.

### Quantitative mass spectrometry

#### TMT labelling and high pH reversed-phase chromatography

100 μg aliquots of isolated mitochondria were digested with trypsin (40:1 protein:protease ratio) overnight at 37°C and labelled using the Tandem Mass Tag (TMT) 10-plex reagent, according to the manufacturer's instructions (Thermo Fisher Scientific, Loughborough, UK), and the labelled samples were pooled. Sample handling and fractionation by high pH reversed-phase chromatography was carried out as previously described (Carrat et al., 2020).

#### Nano-LC mass spectrometry

High pH reverse-phase fractions were further fractionated using an Ultimate 3000 nano-LC system in line with an Orbitrap Fusion Tribrid Mass Spectrometer (Thermo Scientific), as previously described (Carrat et al., 2020).

Raw data files were processed and quantified using Proteome Discoverer software v2.1 (Thermo Scientific) and searched against the UniProt *C. elegans* database (downloaded in December 2018; 27,626 entries) using the SEQUEST algorithm. Peptide precursor mass tolerance was set at 10 ppm, and MS/MS tolerance was set at 0.6 Da. Search criteria included oxidation of methionine (+15.9949) as a variable modification and carbamidomethylation of cysteine (+57.0214) and the addition of the TMT mass tag (+229.163) to peptide N-termini and lysine as fixed modifications (these modifications most likely occurred at some point during sample preparation). Searches were performed with full tryptic digestion, and a maximum of two missed cleavages were allowed. The reverse database search option was enabled, and all data was filtered to satisfy a false discovery rate (FDR) of 5%.

Statistical analysis for the quantitative mass spectrometry was carried out first by log_2_ transforming the data (protein abundance) so that the data followed near normal distribution and skewness was reduced. The log_2_ fold change was then calculated between *nduf-11(RNAi)* and the control samples by subtracting the mean of the former from that of the latter. Unpaired, two-tailed Student's *t*-tests were performed on the transformed data to calculate the *P*-values. The log_2_ fold change and log_10_ of the *P*-values were then plotted on the volcano plots to show the distribution of the data.

### O_2_ consumption

Oxygen consumption was measured by high-resolution respirometry at 25°C in a KCl-based respiration buffer at pH 7.3 (125 mM KCl, 10 mM Tris-HCl, 20 mM MOPS, 2.5 mM KH_2_PO_4_ and 2.5 mM MgCl_2_) (Wojtovich et al., 2008) using a 2 ml chamber of an Oxygraph 2k (Oroboros Instruments, Innsbruck, Austria). 0.25 mg/ml mitochondria in respiration buffer were added to each chamber and equilibrated before the addition of various respiratory substrates (final concentrations as indicated): 10 mM L-malate and 5 mM pyruvate (complex I-linked respiration), or 5 mM succinate plus 1 μM rotenone (complex II-linked respiration). After a stable respiration state 2 was achieved, ADP was added to 1 mM (excess), followed by cytochrome *c* (10 µM). 1 μM oligomycin was used to inhibit ATP synthase and induce a pseudo-state 4. These parameters were based in part on previously published respiration experiments in *C. elegans* (Wojtovich et al., 2008). Respiration rates were calculated as the average value over a 30 s window in DatLab 5 (Oroboros Instruments) and expressed in nmol O_2_/min/mg protein. Respiration states were identified according to Chance and Williams (1955). Statistical significance between groups was determined using a paired two-tailed Student's *t*-test to control for day-to-day variability associated with the isolation protocol and electrode calibration.

### Mitochondrial membrane potential

Membrane potential (ΔΨ) assessment was carried out with the fluorescent potentiometric dye tetramethylrhodamine methyl ester (TMRM; Sigma-Aldrich) working in quench mode (1 µM). Mitochondrial depolarisation causes an increase in fluorescence. Fluorescence was recorded at room temperature with a Varian Cary 50 UV-Vis spectrophotometer (Agilent Technologies, Santa Clara, CA, USA) using 2 ml of KCl respiration medium (see above) supplemented either with 1 µM TMRM, 10 mM L-malate and 5 mM pyruvate (complex I-linked respiration) or with 5 mM succinate plus 1 μM rotenone (complex II-linked respiration). After obtaining a stable signal in the absence of the biological sample, the reaction was started by addition of mitochondria (0.25 mg/ml) and followed by substrates and/or inhibitors added sequentially: coupled respiration was stimulated with the addition of 1 mM ADP (state 3 respiration); ATP synthase was inhibited with 1 μM oligomycin; and maximum depolarisation was achieved using 1 μM CCCP and 1 μM antimycin A.

No attempt was made to calibrate the fluorescent TMRM signal and, thus, relative fluorescent units rather than milli-volts are shown in this work. The averaged signal of each plateau was calculated over 15 s using DatLab 2 in MS-DOS. Statistical significance between groups was determined using a paired two-tailed Student's *t*-test to control for day-to-day variability associated with the isolation protocol.

### Hydrogen peroxide production

ROS production was assayed using the hydrogen peroxide-sensitive fluorescent dye Amplex Red (Thermo Fisher Scientific), as described previously (Andrienko et al., 2016; Wang et al., 2017), and measured every 0.1 s using a Varian Cary 50 UV-Vis spectrophotometer (Agilent Technologies, Santa Clara, CA, USA) at room temperature with manual mixing between additions. Reactions were performed in a 2 ml cuvette in KCl-based respiration medium supplemented either with 10 mM L-malate and 5 mM pyruvate (complex I-linked respiration) or with 5 mM succinate (complex II-linked respiration). After acquiring a baseline for 45 s, mitochondria were added at a concentration of 0.25 mg/ml. After 2 min, inhibitors were sequentially added at 2 min intervals as follows: 1 μM rotenone for inhibition at complex I level, and 1 μM antimycin A for inhibition at complex III level. A linear rate was observed after each addition and during the 2 min timeframe. Fluorescence at specific timepoints, namely 15 s before the next addition, was used to characterise each state and compare ROS production between groups. Statistical significance between groups was determined using a paired two-tailed Student's *t*-test to control for day-to-day variability associated with the isolation protocol.

### Biochemical enzymatic activities

Spectrophotometric analysis of respiratory complex I and II and citrate synthase activities were performed at 25°C using a CLARIOstar Plus (BMG Labtech).

#### Complex II activity

The assay used was adapted from a previous report using mammalian samples (Munujos et al., 1993). Briefly, mitochondria were preincubated at 5 mg/ml in a solution of 3 mM succinate in 10 mM potassium phosphate at pH 7.4 for 30 min at 25°C, then lysed by adding an equal volume of 0.2% Triton X-100 followed by three freeze–thaw cycles. Complex II activity was determined by measuring reduction of DCPIP (2,6-dichlorophenolindophenol) at 600 nm (*ε*=19.1 mM^−1^ cm^−1^; Birch-Machin and Turnbull, 2001) in a reaction mixture composed of 50 mM potassium phosphate (pH 7.0), 2 mM KCN, 0.1 mM DCPIP, 2 µg/ml rotenone, 2 µg/ml antimycin A and lysed mitochondria (0.25 mg/ml). A baseline rate of DCPIP reduction was measured for 3 min before addition of succinate (16 mM), followed by measurements for 2 min. Finally, CoQ_1_ (50 µM) was added, and measurements continued for another 20 min. Activity was calculated by fitting the linear slope of the initial 9 min after CoQ1 addition in the presence of succinate.

#### Complex I activity

Complex I activity was determined by following reduction of a dye (from Abcam kit ab109721) at 450 nm (*ε*=25.9 mM^−1^ cm^−1^), which increases in absorbance relative to oxidation of NADH to NAD^+^. Lysed mitochondria were diluted to 33.3 µg/ml in 1× reaction buffer (Abcam kit ab109721) containing 1× dye. Baseline absorbance of the dye was measured at 450 nm for 2.5 min before starting the reaction with the addition of NADH (2 mM). Activity was calculated by fitting the linear slope of the initial 9 min after NADH addition.

#### Citrate synthase activity

The DTNB method (Srere, 1963) was used to assay the activity of citrate synthase in lysed mitochondria (10–25 µg/ml) in a mixture of 100 mM Tris-HCl (pH 7.4) and DTNB (0.1 mM). Absorbance at 412 nm (*ε*=14.15 mM^−1^ cm^−1^) was measured for 2 min before adding acetyl-CoA (50 µM) and allowing stabilisation before starting the reaction by adding oxaloacetate (100 µM). Increasing absorbance, corresponding to the formation of CoASH during citrate synthesis, was followed for 20 min. Activity was calculated by fitting the linear slope of the initial 1.5–2.5 min after oxaloacetate addition in the presence of acetyl-CoA.

### LDS-PAGE

Pre-cast Bolt gels and buffers (Thermo Fisher Scientific) were used. Whole worm protein samples consisted of ∼50 adult worms in 15 μl of M9 buffer mixed with an equal volume of 4× Bolt LDS NuPAGE sample buffer containing 250 mM 1,4-dithiothreitol (DTT). Mitochondrial samples contained 50 μg of protein diluted 1:4 with 4× Bolt LDS NuPAGE sample buffer containing 250 mM DTT. All samples were incubated at 60°C for 25 min and centrifuged for 1 min at 14,500 ***g*** before electrophoresis on 4–12% Bolt Bis-Tris Plus gels at 150 V (constant) in Bolt MOPS running buffer for ∼30 min. Gels were transferred to 0.45 μm PVDF membranes (Millipore) at a 25 V (constant) for 10 min using a Pierce semi-dry G2 Fast Blotter (Thermo Fisher Scientific) according to the manufacturer's instructions.

### Western blotting

Membranes were incubated in BLOTTO [5% milk in TBS-T (50 mM Tris-HCl, pH 7.6, 150 mM NaCl and 0.05% Tween-20)] for ∼1 h at room temperature. Primary antibodies were diluted in BLOTTO and incubated for 1 h at room temperature with constant shaking. Membranes were washed with TBS-T for 10 min before incubating for 1 h at room temperature with a secondary antibody conjugated to goat anti-mouse HRP (NB7539; Novus Biologicals, Centennial, CO, USA) with a titre of 1:10,000 in BLOTTO. Membranes were washed three times with TBS-T for 10 min each before incubation with Amersham ECL detection reagent (GE Healthcare) and chemiluminescent detection using the Li-COR Odyssey FC imaging system (LI-COR Biosciences). Alternatively, secondary antibodies conjugated with fluorescent dyes were directly visualised at 700 or 800 nm (anti-mouse 800, Invitrogen, SA5-10172; anti-rabbit 680, ThermoFisher, 35569). Western blot quantification was performed using LI-COR image studio. Antibodies and titres used in this study include mouse anti-NDUFS3 [17D95] mAb (Abcam, ab14711) at 1:1000 and mouse anti-ATP5A [15H4C4] mAb (Abcam, ab14748) at 1:1000. A rabbit polyclonal antibody recognising a N-terminal peptide sequence of NDUF-11 (CEL-B0491.5; GHGEEPLTATYKTQR-[C]-amide) was produced by Cambridge Research Biochemicals (Billingham, UK) and used at a titre of 1:500. Statistical significance between groups was determined using an unpaired two-tailed Student's *t*-test.

### BN-PAGE

Previously prepared mitochondria (1 or 2 mg) were thawed and pelleted at 14,500 ***g*** for 15 min at 4°C, then resuspended in solubilisation buffer (50 mM NaCl, 10% glycerol, 1 mM PMSF and 20 mM Tris-HCl pH 7.5) supplemented with either 10 mg/ml digitonin (Millipore-Sigma, cat. 300410) or 5 mg/ml Triton X-100 (Merck, X100). Solubilisation was carried out at 4°C for 20 min with mild agitation, followed by centrifugation at 14,500 ***g*** for 15 min at 4°C. Solubilised proteins in a 25 μl volume were prepared and separated by electrophoresis on NativePAGE 3–12% Bis-Tris gels (Thermo Fisher Scientific) and NuPAGE Tris-acetate buffer (Thermo Fisher Scientific), according to the manufacturer's instructions, at 30 V (constant) for ∼16 h or until the dye front reached the gel bottom. Gels were either stained with Coomassie G-250 (50% methanol, 10% acetic acid and 0.5% Coomassie G-250) and de-stained (10% methanol; 10% acetic acid), or silver stained using the Silver Quest kit (Thermo Fisher Scientific).

### In-gel respiratory complex

In-gel identification of respiratory complexes separated by BN-PAGE was performed using previously published methods (Smet et al., 2006; Zerbetto et al., 1997). Briefly, BN-PAGE gels were incubated at room temperature on an orbital shaker at 60 r.p.m. with complex I activity buffer (50 mM potassium phosphate buffer pH 7.4, 0.1 mg/ml NADH and 0.2 mg/ml nitro blue tetrazolium chloride). Incubation was carried out for ∼1 h.

### Two-dimensional BN-PAGE–LDS-PAGE

A lane from a BN-PAGE gel was trimmed to fit the loading area of a 2nd dimension LDS-PAGE gel and incubated in 10 ml of reducing solution (1× Bolt LDS NuPAGE sample buffer with 250 mM DTT) for 30 min with agitation. The gel lane was then inserted into the well of a pre-cast 4–12% NuPage Bis-Tris 1-well gel. The well was overlaid with 1× Bolt LDS buffer and electrophoresed at 150 V (constant) for 40 min or until the dye front reached the gel bottom. The gel was either silver stained (see above) to visualise total protein or used for western blot analysis to detect specific proteins.

### Statistical analysis

Data were assessed for normality using the Kolmogorov–Smirnov test with Dallal–Wilkinson–Lillie for correction. Overall, analysed data were normally distributed. Since group sample sizes were equal and the parametric statistical tests applied are robust for moderate deviations from homoscedasticity, parametric tests were always applied (Zar, 1999).

The statistical tests used in this study are described in each appropriate section. Statistical analyses were performed using Graph Pad Prism version 7.0 and/or 8.0 (GraphPad Software, Inc., San Diego, CA, USA). Volcano plots were made in VolcanoR (Naumov et al., 2017 preprint).

## Supplementary Material

Supplementary information

Reviewer comments
