# Supplementary material for: Maintenance of complex I and its supercomplexes by NDUF-11 is essential for mitochondrial structure, function and health
Source: J Cell Sci. 2021 Jul 9;134(13):jcs258399. doi: 10.1242/jcs.258399 (PMC8277142; doi:10.1242/jcs.258399)
Supplement: Supplementary information [file joces-134-258399-s1.pdf]

Table S1. Brood count after *cel-NDUFA11* silencing.

	2nd generation developmental arrest					
	Total Brood Count [a]	L1	L2	L3	L4	Adult
Wild type (n=6)	282 ± 2.70 (n=6)	-	-	-	-	100 ± 0%
Cel-NDUFA11(*RNAi*)	1.9 ± 0.62 *** (n=10)	12.4 ± 6.05%	87.6 ± 6.05%	-	-	-

[a] Mean ± SEM, 6 (control) and18 (cel-NDUFA11(RNAi)) independent experiments, for the Developmental Stage analysis. Differences between group on the Brood Count analysis were assess by an Unpaired t-Test. ***, p < 0.001.

Table S2. Human homologues of *C. elegans* proteins highlighted in main Fig. 3c and supplementary Fig. S2. Orthologues were checked against the Alliance of Genome Resources database, release 4.0.0.

Location in the text	*C. elegans* Acc. Numb	Human Homologue
Fig 3c	pgam-5	PGAM5
Fig 3c	tofu-2	N/A
Fig 3c	cyp-33c8	CYPIIA13
Fig 3c	acs-2	ACSF2
Fig 3c	CELE_W05F2.3	N/A
Fig 3c	rme-2	N/A
Fig 3c	CELE_F17C11.13	N/A

Journal of Cell Science • Supplementary information

Fig 3c	cpg-1	N/A
Fig 3c	B0205.13	N/A
Fig 3c	nduf-2.2	NDUFS2
Fig S2 – Complex I	nuo-1	NDUFV1
Fig S2 – Complex I	F53F4.10	NDUFV2
Fig S2 – Complex I	nuo-5	NDUFS1
Fig S2 – Complex I	gas-1	NDUFS2
Fig S2 – Complex I	nduf-2.2	NDUFS2
Fig S2 – Complex I	nuo-2	NDUFS3
Fig S2 – Complex I	nduf-7	NDUFS7
Fig S2 – Complex I	T20H4.5	NDUFS8
Fig S2 – Complex I	nduo-1	ND1
Fig S2 – Complex I	nduo-2	ND2
Fig S2 – Complex I	nduo-3	ND3
Fig S2 – Complex I	nduo-4	ND4
Fig S2 – Complex I	ndfl-4	ND4L
Fig S2 – Complex I	nduo-5	ND5
Fig S2 – Complex I	nduo-6	ND6
Fig S2 – Complex I	lpd-5	NDUFS4
Fig S2 – Complex I	nduf-5	NDUFS5
Fig S2 – Complex I	nduf-6	NDUFS6
Fig S2 – Complex I	CELE_Y63D3A.7	NDUFA2
Fig S2 – Complex I	C33A12.1	NDUFA5
Fig S2 – Complex I	nuo-3	N/A
Fig S2 – Complex I	CELE_F45H10.3	NDUFA7
Fig S2 – Complex I	CELE_Y54F10AM.5	NDUFA8
Fig S2 – Complex I	CELE_Y53G8AL.2	NDUFA9
Fig S2 – Complex I	nuo-4	NDUFA10
Fig S2 – Complex II	sdha-1	SDHA
Fig S2 – Complex II	sdha-2	SDHA
Fig S2 – Complex II	sdhb-1	SDHB
Fig S2 – Complex II	mev-1	SDHC
Fig S2 – Complex II	sdhd-1	SDHD
Fig S2 – Complex III	ctb-1	MT-CYB
Fig S2 – Complex III	cyc-1	CYC1
Fig S2 – Complex III	isp-1	UQCRFS1
Fig S2 – Complex III	ucr-1	UQCRC1
Fig S2 – Complex III	ucr-2.1	UQCRC2
Fig S2 – Complex III	ucr-2.2	UQCRC2
Fig S2 – Complex III	ucr-2.3	UQCRC2
Fig S2 – Complex III	CELE_T27E9.2	UQCRH
Fig S2 – Complex III	CELE_T02H6.11	UQCRB
Fig S2 – Complex III	CELE_R07E4.3	UQCRQ
Fig S2 – Complex III	CELE_F45H10.2	UQCRQ
Fig S2 – Complex III	C14B9.10	UQCR10

Fig S2 – Complex III	ucr-11	UQCR11
Fig S2 – Complex III	ddl-3	TTC19
Fig S2 – Complex IV	ctc-1	MT-CO1
Fig S2 – Complex IV	ctc-2	MT-CO2
Fig S2 – Complex IV	ctc-3	MT-CO3
Fig S2 – Complex IV	cox-4	COX4I1
Fig S2 – Complex IV	cox-5A	COX5A
Fig S2 – Complex IV	cox-5b	COX5B
Fig S2 – Complex IV	cox-6A	COX6A1/2
Fig S2 – Complex IV	cox-6b	COX6B1/2
Fig S2 – Complex IV	cox-6c	COX6C
Fig S2 – Complex IV	cox-7c	COX7C
Fig S2 – Complex V	atp-1	ATP5F1A
Fig S2 – Complex V	atp-2	ATP5F1B
Fig S2 – Complex V	CELE_Y69A2AR.18	ATP5F1C
Fig S2 – Complex V	F58F12.1	ATP5F1D
Fig S2 – Complex V	hpo-18	ATP5F1E
Fig S2 – Complex V	R05D3.6	N/A
Fig S2 – Complex V	Y82E9BR.3	ATP5MC1/2/3
Fig S2 – Complex V	CELE_R04F11.2	ATP5ME
Fig S2 – Complex V	R53.4	ATP5MF
Fig S2 – Complex V	asg-1	ATP5MG
Fig S2 – Complex V	asg-2	ATP5MG
Fig S2 – Complex V	atp-6	MT-ATP6
Fig S2 – Complex V	asb-1	ATP5PB
Fig S2 – Complex V	asb-2	ATP5PB
Fig S2 – Complex V	atp-5	ATP5PD
Fig S2 – Complex V	atp-4	ATP5PF
Fig S2 – Complex V	atp-3	ATP5PO
Fig S2 – Complex V	mai-2	ATP5IF1
Fig S2 – SLCs	C33F10.12	SLC25A3
Fig S2 – SLCs	slc-25a18.2	SLC25A18/22
Fig S2 – SLCs	hpo-12	SLC25A19
Fig S2 – SLCs	F17E5.2	SLC25A23/24/25/41
Fig S2 – SLCs	K11H3.3	SLC25A1
Fig S2 – SLCs	slc-25a10	SLC25A10
Fig S2 – SLCs	misc-1	SLC25A11
Fig S2 – SLCs	C47E12.2	SLC25A4/5/6/31
Fig S2 – SLCs	K02F3.2	SLC25A12/13
Fig S2 – SLCs	CELE_T10F2.2	SLC25A2/15
Fig S2 – SLCs	ant-1.4	SLC25A4/5/6/31
Fig S2 – SLCs	mfn-1	SLC25A28/37
Fig S2 – SLCs	CELE_F01G4.6	SLC25A3
Fig S2 – SLCs	slc-25a42	SLC25A42

Fig S2 – SLCs	slc-25a18.1	SLC25A18/22
Fig S2 – SLCs	slc-25a21	SLC25A21
Fig S2 – SLCs	CELE_F25B4.7	SLC25A4/5/6
Fig S2 – SLCs	slc-25a26	SLC25A26
Fig S2 – SLCs	CELE_T09F3.2	SLC25A33/36
Fig S2 – SLCs	dif-1	SLC25A20
Fig S2 – SLCs	slc-25a32	SLC25A32

Table S3. Oligonucleotides used in this study.

Lab Sequence ID	Sequence
PK1801	TCTTGGGTCTCTGCGTTTTATACG
PK1802	AAACCGTATAAAACGCAGAGACCC
PK1803	TCTTGCCTGGCTCCTTCCACCAGT
PK1804	AAACACTGGTGGAAGGAGCCAGGC
PK1805	GTTTTCCAGAACGCGTGACAGAGTCTTCAAGTCCTGGCTCCTTCCACCAGTTCATCT ATGGCGGTCAATGGTTCTTCTCCGTGGCCCATTTTACCTGAAAATCGATTG

Journal of Cell Science • Supplementary information

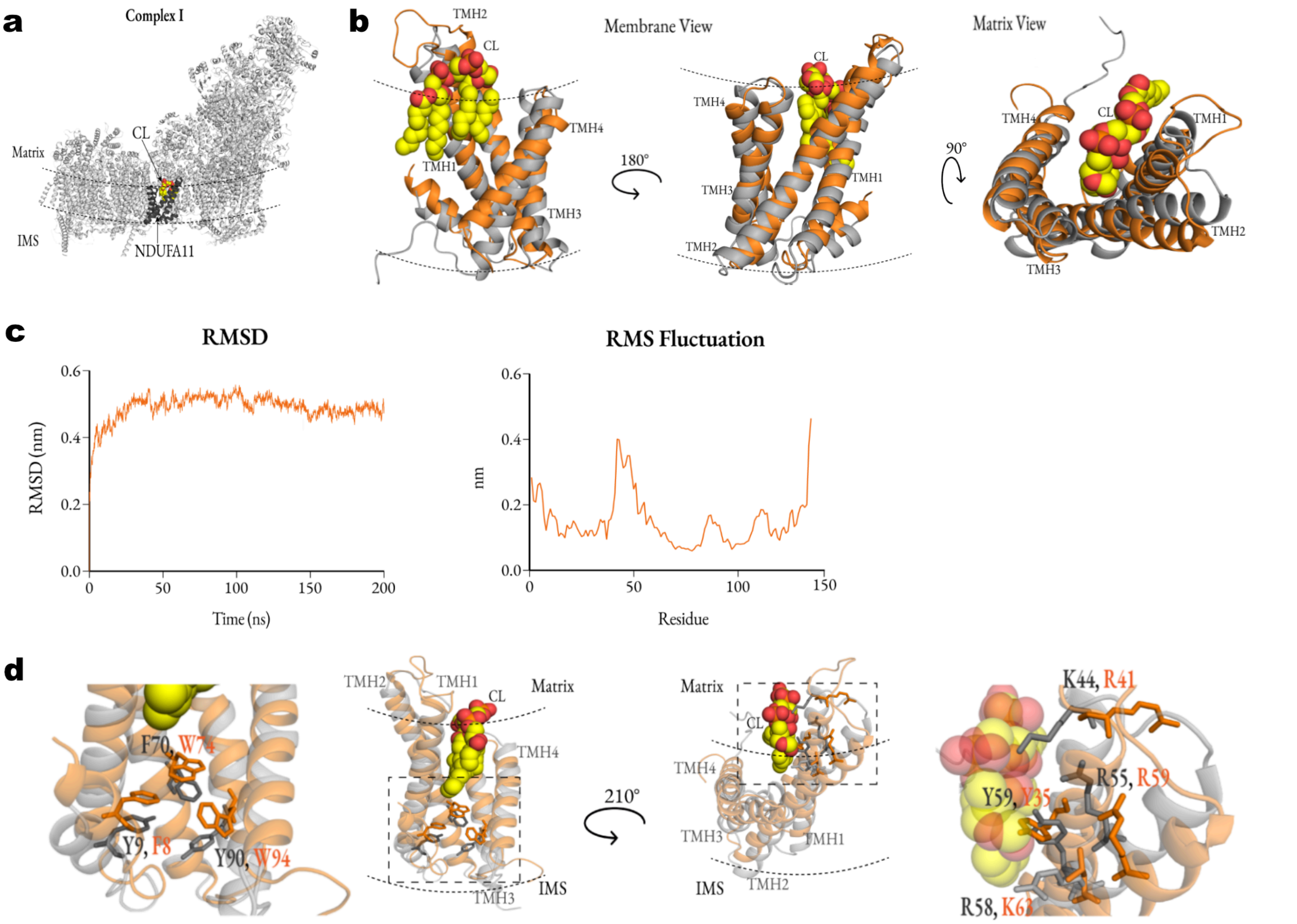

Figure S1. (a) Human NDUFA11 subunit (darker grey) on Complex I (light grey; porcine, pdb: 5GUP, [19]) used for the homology model and derived homology models of *C. elegans* NDUF-11 **(b** and **d)**. **(b)** Three orientations of the *C. elegans* NDUF-11 model (orange) overlaid with NDUFA11 structure from 5GUP (grey). **(c)** Left: RMSD plot of the *C. elegans* NDUF-11 protein backbone over 200 ns of atomistic simulation. The protein is very stable, considering that the input is a homology model. Right: RMS fluctuation of each residue over the simulation, fitting to the input structure. The main protein is very stable, with a flexible loop between TMH1 and TMH2, and a flexible C-terminus. **(d)** Cardiolipin interacting residues shown in membrane view of different orientations; zoomed regions of the dotted box are shown on the side of the central panels. Residues labelled in the figure correspond to the NDUFA11 structure (grey) and NDUF-11 homology model (orange). All structures were created in PyMOL and assembled in a photo editor; the dotted lines represent the membrane border. Abbreviations: CI – Complex I, $CIII_2$ – dimer of Complex II, CIV – Complex IV, CL – cardiolipin, IMS – intermembrane space, THM – transmembrane helix.

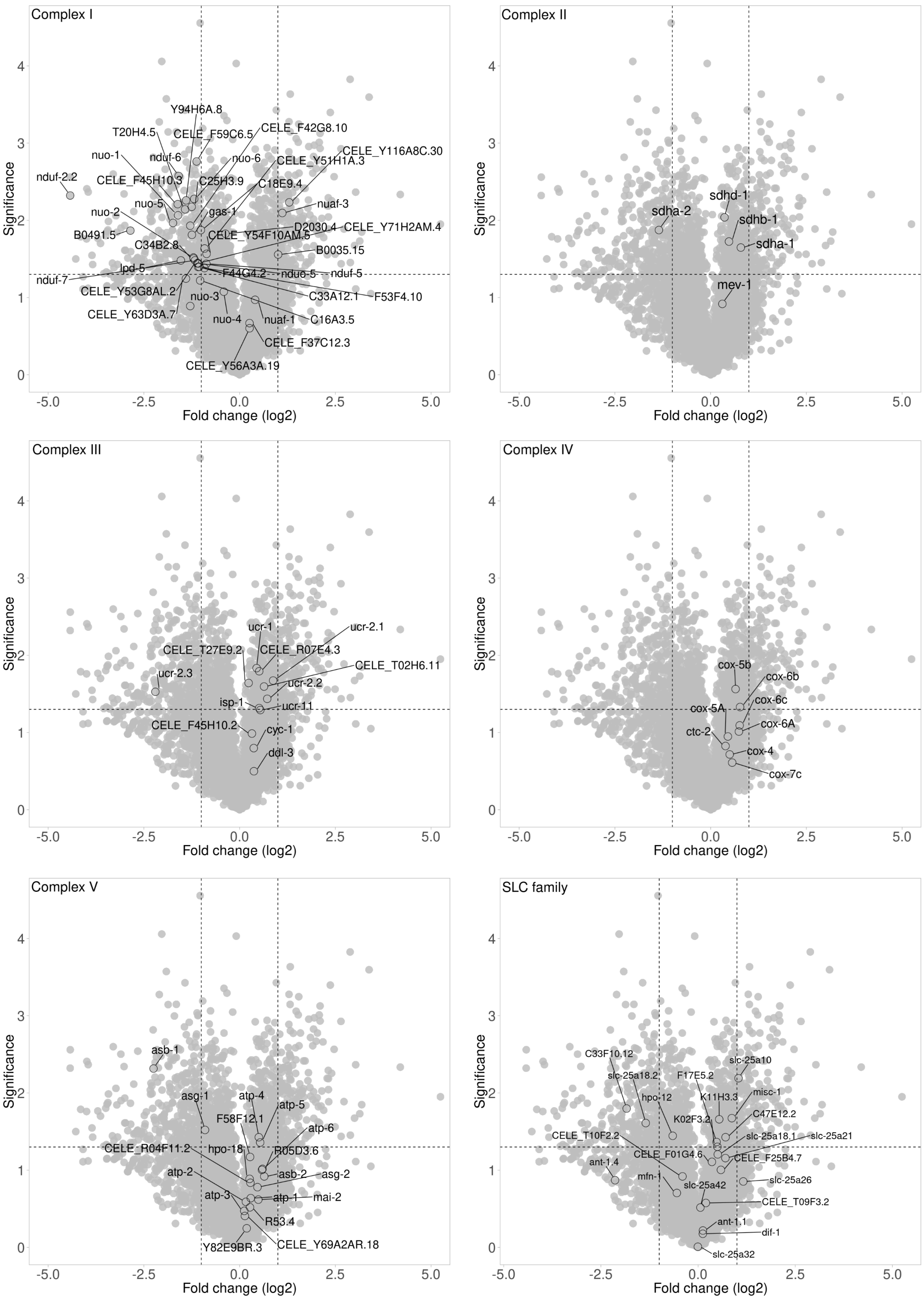

Journal of Cell Science • Supplementary information

Figure S2. Volcano plots showing mass spec data from isolated mitochondrial fractions as shown in Fig. 3c. In each panel, labelled data represent subunits of OXPHOS complexes as shown on the top left corner of each graph; and, members of the solute carrier superfamily (SLC). The corresponding human orthologues are shown in Supp. Table S2. Vertical dashed lines represent the user-defined threshold in expression levels while horizontal dash lines represent the p-value of 0.05. Therefore, the most upregulated genes are towards the right, the most downregulated genes are towards the left, and the most statistically significant genes are towards the top. Plots are in $\log_2$-$\log_2$ scale and were generated using VolcanoR.

Journal of Cell Science • Supplementary information

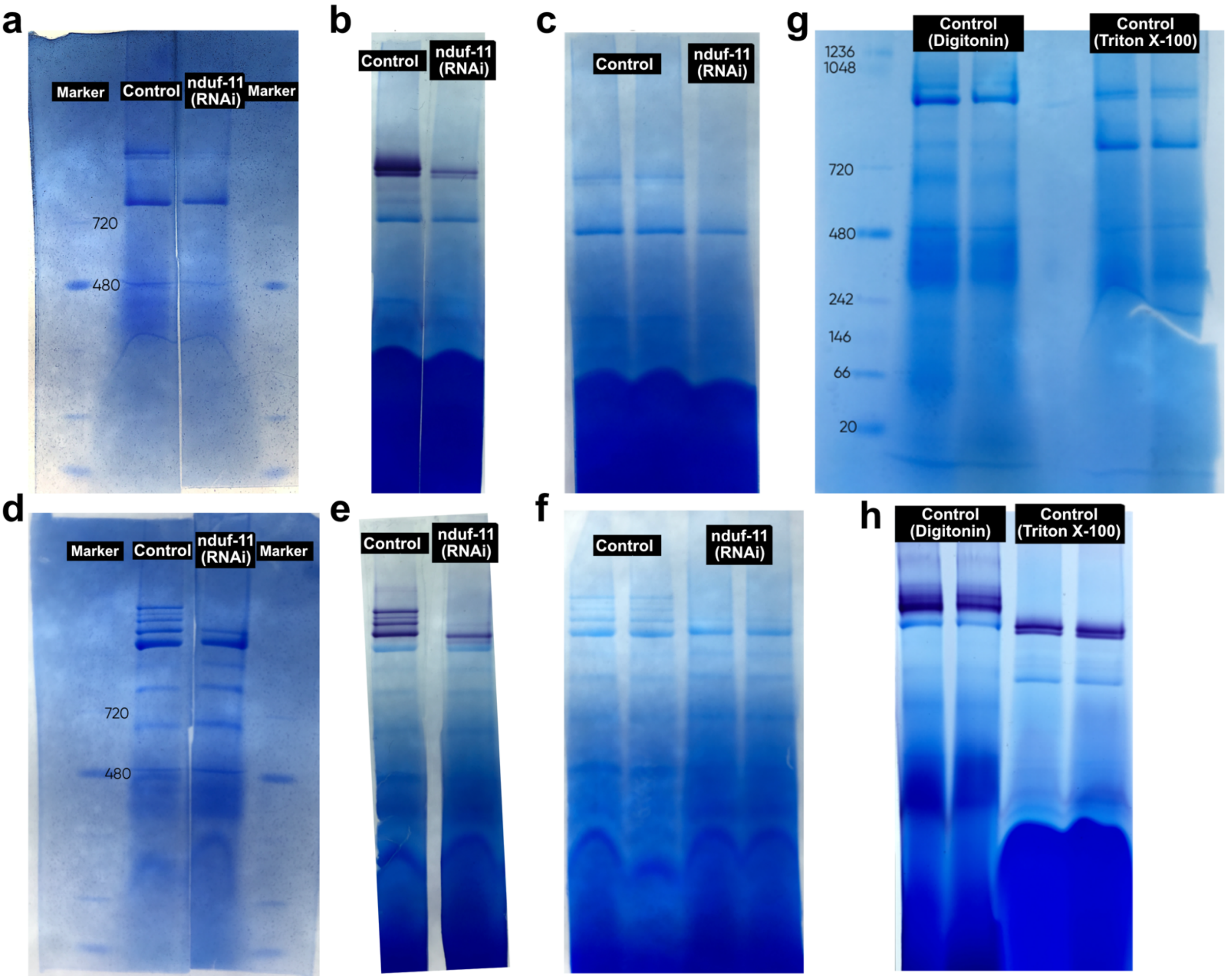

Figure S3. Supporting information for main figure 4 showing uncropped gels (**a, b, d, e**) for Coomassie stain (**a, d**) and *in gel* Complex I activity (**b, e**), for mitochondria factions solubilised with Triton X-100 (**a, b**) or digitonin (**d, e**). Samples were run in the same gel under the conditions but lanes were cut out in order to stain for different purposes. An example of uncut gels (unstained) is shown on panels c and f and shows that the pattern of the major bands is unchanged after RNAi treatment. The position of the strongest band(s) was used to align the cut gels. Side by side comparison of Triton X-100 vs Digitonin treated samples in uncut gels is shown for the Control group (**g, h**), for Coomassie stain (**g**) and in gel Complex I activity (**h**).

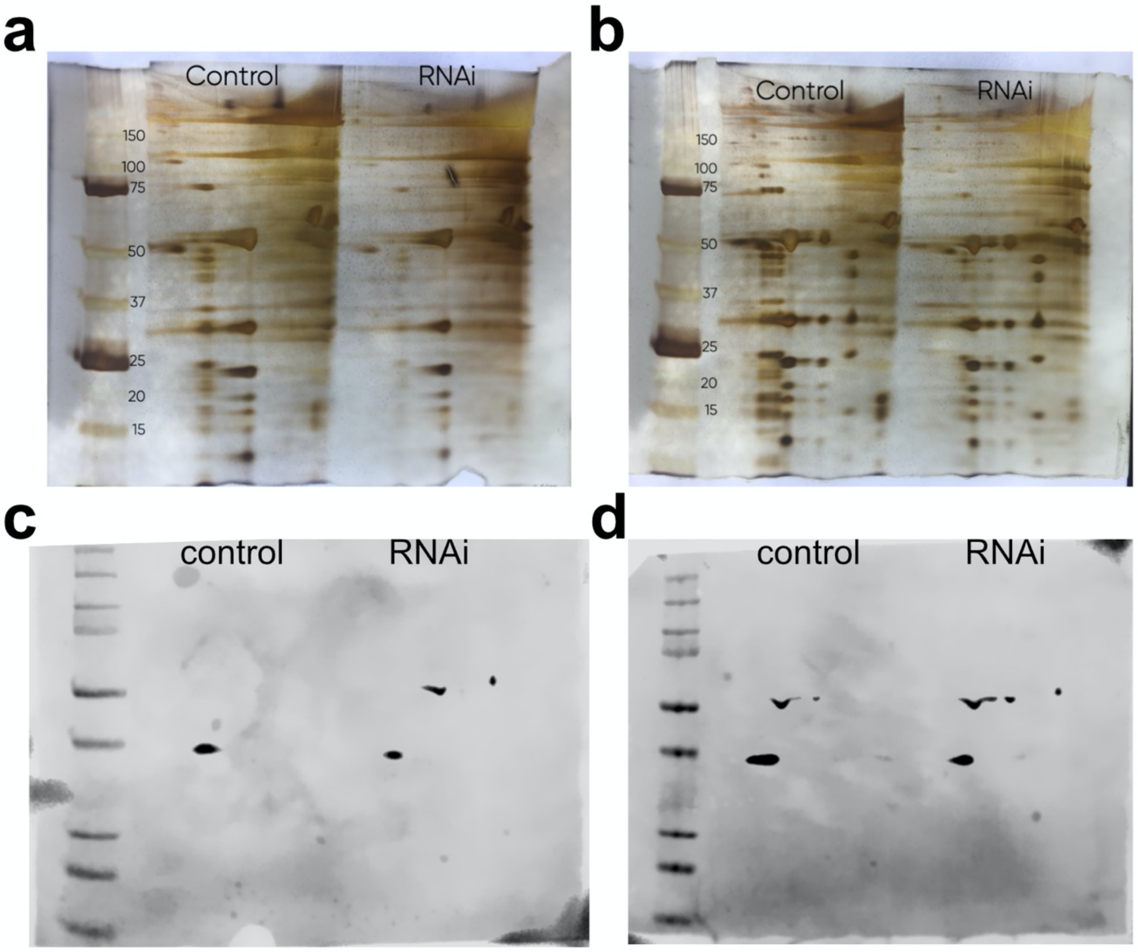

Figure S4. Supporting information for main figure 4 showing uncropped gels (**a**, **b**) and membranes (**c**, **d**). Detection on **a** and **b** was by Silver stain and fluorescent-based antibodies on **c** and **d**. Panels **a** and **c** show Triton X-100 treated samples while **b** and **d** shown the digitonin-treated ones.

Journal of Cell Science • Supplementary information

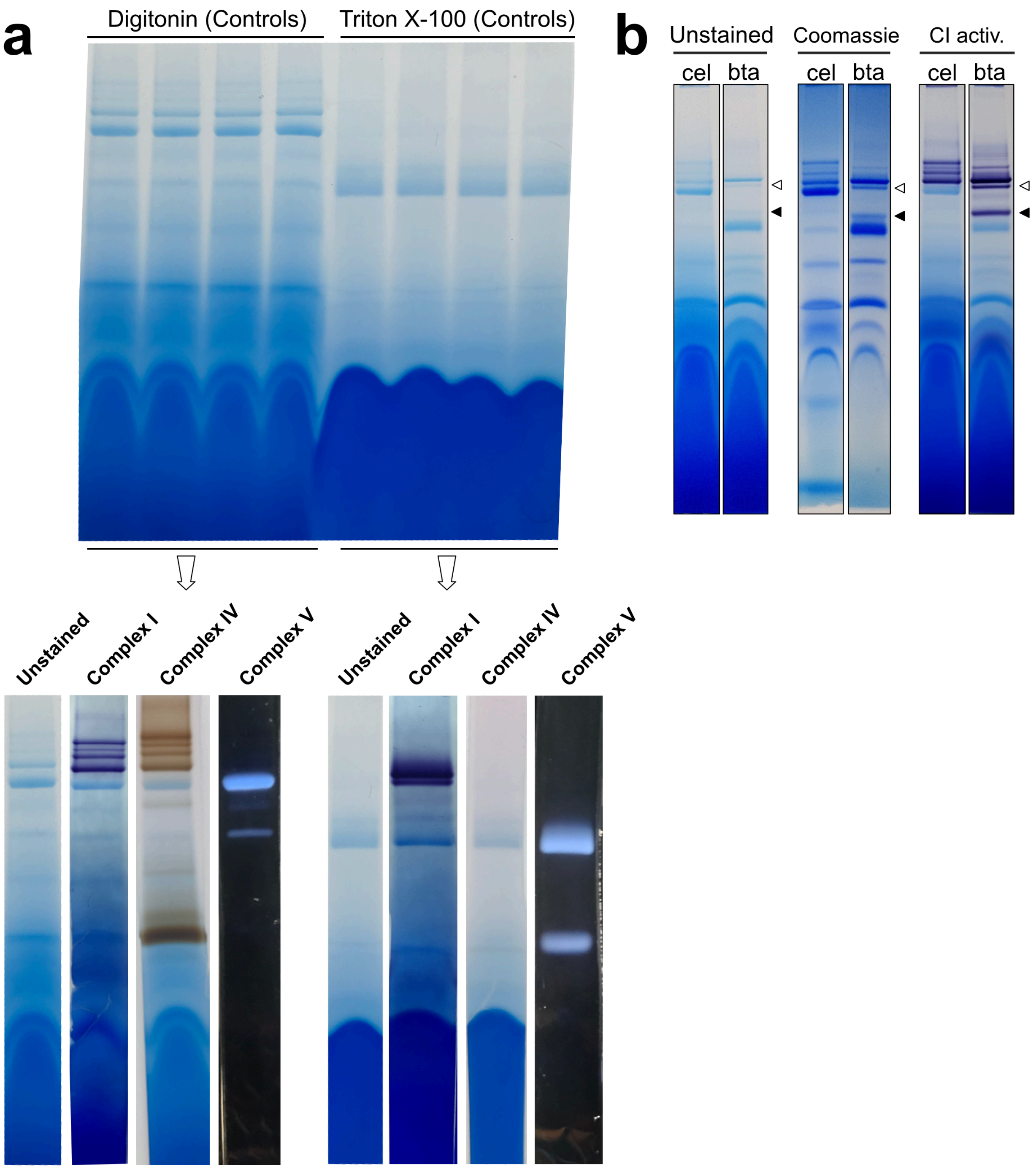

Figure S5. Supporting information for main figure 4 showing the identification of respiratory complexes in BN gels by specific in-gel activity assays for Complex I, IV and V (**a**). Samples shown are from Control group only. Gel lanes were cut vertically and used for each individual biochemical assay; image alignment was done offline. Panel **b** shows isolated mitochondria from worm (cel) and bovine (bta) solubilised with digitonin and stained with Coomassie or by in-gel Complex I activity. The **black arrowhead** highlights monomeric Complex I in bovine mitochondria which appears to be absent in the worm sample. The **white arrowhead** highlights a Complex I-positive super-complex entity in bovine mitochondria that is not observed in N2 control worm mitochondria. This is usually attributed to the super-complex core unit CI:III2. Interestingly, a similar band pattern is observed in nduf-11 RNAi mitochondria (see Fig. 4c, arrowhead; and, Fig. S3e).

Journal of Cell Science • Supplementary information

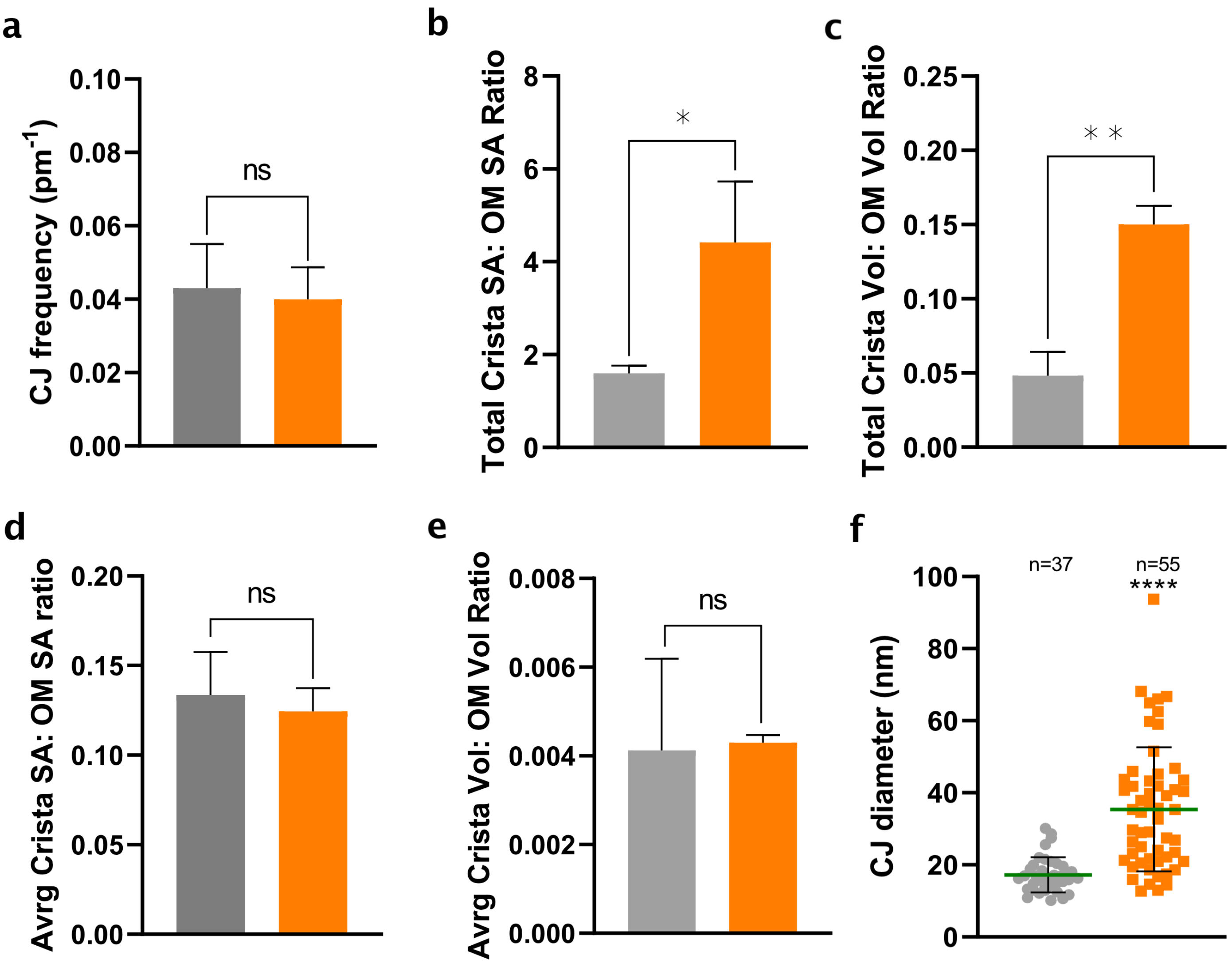

Figure S6. Effect of NDUF-11 knockdown on the total and average crista surface areas and volumes, and CJ frequency and diameter. (**a**) Effect of *nduf-11* knockdown on CJ frequency. The number of CJs in the segmented models from main Fig. 6a-f were counted, and normalised to the outer membrane surface area. **(b,c)** Total crista surface area and volume presented as a ratio to outer membrane surface area and volume, respectively. There is an increase in the total surface area and volumes of crista membranes relative to mitochondrial size for the NDUF-11 knockdown. **(d,e)** Average crista surface area and volume presented as a ratio to the outer membrane surface area and volume, respectively. Mesh surface area and volume inside the mesh of each membrane from mitochondrial reconstructions shown in main Fig. 6a-h were calculated computationally (n=3 for each condition, and n = 37 (wild-type) and 105 (NDUF-11) for total crista analysed). There is no significant change in the average surface area and volume per crista membrane relative to mitochondrial size, representative of a mix of fused and fragmented cristae in the NDUF-11 knockdown. **(f)** Effect of NDUF-11 knockdown on distribution of CJ diameters. CJ diameter was measured from mitochondrial reconstructions. The diameters of 37 junctions from three wild-type mitochondria shown in main Fig. 6a-c, and 55 junctions from three nduf- 11(RNAi) mitochondria shown in main Fig. 6d-f were plotted individually.

Unpaired, parametric t-tests were used to calculate all significance values: * $p \leq 0.05$, ** $p \leq 0.01$. Error bars: SEM in panel b-c, SD in others.

Journal of Cell Science • Supplementary information

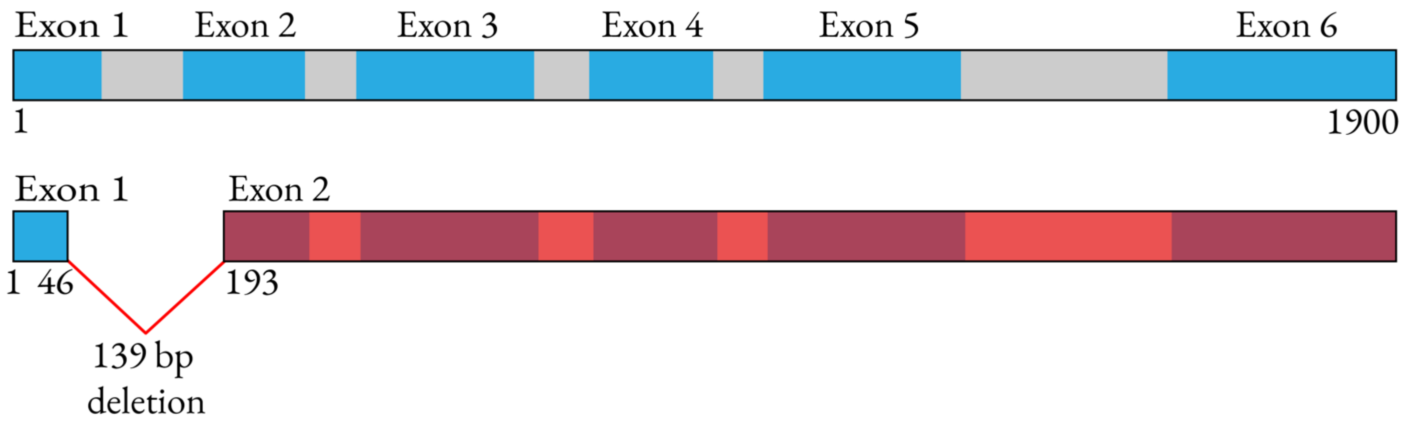

Figure S7. *nduf-11*(*cr51*) CRISPR-Cas9 mutant - schematic representation of the CRISPR mutation introduced to *nduf-11*, leading to a deleted region in between position 46 and 193 and thus a frameshift mutation which perturbs the rest of the sequence represented in red.

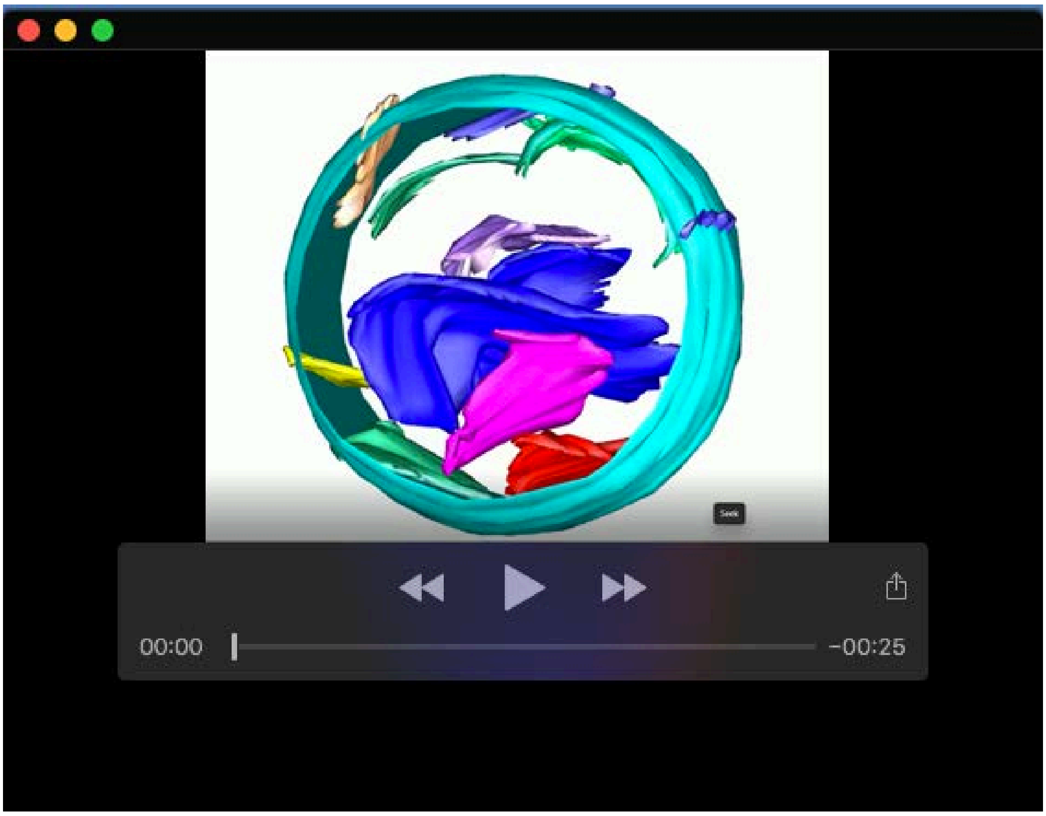

Movie 1. Movie showing a 360° rotation about the y-axis of segmentations from Fig. 6a (control sample). The outer membrane is hidden to display crista junction morphology. An image sequence of 100 PGN files was collected in IMOD, and sequence montaged into 10fps mp4 file in Image

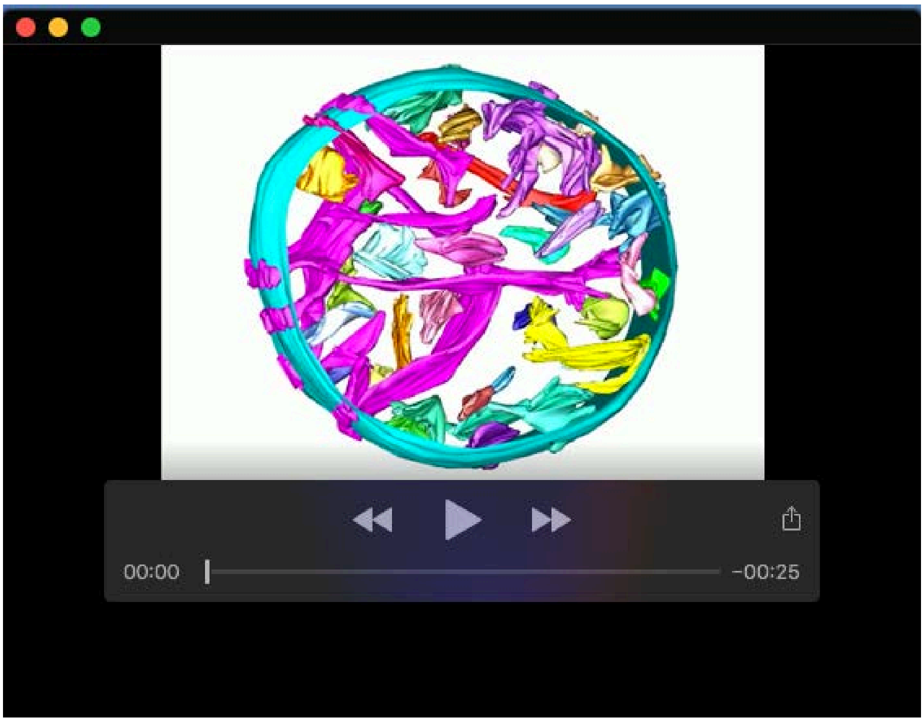

Movie 2. Movies showing a 360° rotation about the y-axis of segmentations from Fig. 6f (RNAi sample). The outer membrane is hidden to display crista junction morphology. An image sequence of 100 PGN files was collected in IMOD, and sequence montaged into 10fps mp4 file in Image

Journal of Cell Science • Supplementary information
