# Supplementary material for: Maintenance of complex I and its supercomplexes by NDUF-11 is essential for mitochondrial structure, function and health
Source: J Cell Sci. 2021 Jul 9;134(13):jcs258399. doi: 10.1242/jcs.258399 (PMC8277142; doi:10.1242/jcs.258399)
Supplement: Reviewer comments [file joces-134-258399_review_history.pdf]

Journal of Cell Science

Maintenance of complex I and its supercomplexes by NDUF-11 is essential for mitochondrial structure, function and health

Amber Knapp-Wilson, Gonçalo C. Pereira, Emma Buzzard, Holly C. Ford, Andrew Richardson, Robin A. Corey, Chris Neal, Paul Verkade, Andrew P. Halestrap, Vicki A. M. Gold, Patricia E. Kuwabara and Ian Collinson
DOI: 10.1242/jcs.258399

Editor: Michael Way

Review timeline
Original submission: 12 January 2021
Editorial decision: 29 January 2021
First revision received: 14 May 2021
Editorial decision: 25 May 2021
Second revision received: 27 May 2021
Accepted: 28 May 2021

Original submission

<u>First decision letter</u>

MS ID#: JOCES/2021/258399

MS TITLE: Maintenance of complex I and its super-complexes for mitochondrial structure, function and health

AUTHORS: Amber Knapp-Wilson, Goncalo C Pereira, Emma Buzzard, Andrew P Richardson, Robin A Corey, Chris Neal, Paul Verkade, Andrew Halestrap, Vicki AM Gold, Patricia E Kuwabara, and Ian Collinson
ARTICLE TYPE: Research Article

We have now reached a decision on the above manuscript.

To see the reviewers' reports and a copy of this decision letter, please go to: https://submit-jcs.biologists.org and click on the 'Manuscripts with Decisions' queue in the Author Area. (Corresponding author only has access to reviews.)

As you will see, both reviewers raise a number of criticisms and questions that prevent me from accepting the paper at this stage. They suggest, however, that a revised version might prove acceptable, if you can address their concerns and comments many of which are clarifications. If you think that you can deal satisfactorily with the criticisms on revision, I would be pleased to see a revised manuscript.

We are aware that you may be experiencing disruption to the normal running of your lab that makes experimental revisions challenging. If it would be helpful, we encourage you to contact us to discuss your revision in greater detail. Please send us a point-by-point response indicating where you are able to address concerns raised (either experimentally or by changes to the text) and where you will not be able to do so within the normal timeframe of a revision. We will then provide further guidance. Please also note that we are happy to extend revision timeframes as necessary.

© 2021. Published by The Company of Biologists under the terms of the Creative Commons Attribution License (https://creativecommons.org/licenses/by/4.0/).

Please ensure that you clearly highlight all changes made in the revised manuscript. Please avoid using 'Tracked changes' in Word files as these are lost in PDF conversion.

I should be grateful if you would also provide a point-by-point response detailing how you have dealt with the points raised by the reviewers in the 'Response to Reviewers' box. Please attend to all of the reviewers' comments. If you do not agree with any of their criticisms or suggestions please explain clearly why this is so.

Reviewer 1

Advance summary and potential significance to field

Knapp-Wilson et al describes a C. elegans model for the disturbance of complex I and supercomplex assembly and/or their structural integrity. The authors identify the C. elegans homologue for subunit NDUFA11, which structurally forms contact sites between complexes I and III in mitochondrial respiratory supercomplexes. The gene knockout of this subunit was lethal, but a strong knockdown was viable and investigated here. The content of intact complex I was much reduced, and there appear to be no stable complex I-containing supercomplexes present. The consequences of these primary effects are on mitochondrial coupling, striking changes to mitochondrial morphology, and a series of metabolic compensatory shifts. Despite these mitochondrial and metabolic perturbations, the animals experienced a longer lifespan than their WT counterparts, but their progeny failed to develop to maturity, indicating a lack of metabolic plasticity under high mitochondrial demands.

This study represents a well-designed and careful body of work that advances knowledge of the effects of complex I and SC dysfunction on mitochondria and on animal health, presented clearly and discussed in an unbiased manner.

Comments for the author

1. Could the authors comment on whether NDUF-11 is essential, or just beneficial for complex I to otherwise reach maturity (with the flavoprotein domain attached)? The possibilities in the knockdown are A) all of the intact cxI observed has the residual NDUF-11, but the quantity of mature complex I is limited to the supply of NDUF-11 B) some of the complex I observed does not have NDUF-11 but otherwise reaches maturity and could therefore be catalytically and structurally compromised or C) none of the mature complex I contains NDUF-11. As the g/m/adp rate of oxygen consumption looks to be ~ 20% of WT, this is consistent with option A) because as long as the catalytic rate limiting step has not changed, it should be proportional to the amount of complex I present. On the other hand, it is not clear from the monomeric complex I observed on in-gel activity on the BN PAGE whether or not the mass has shifted (see below). The lack of SC in digitonin does not seem to inform on this because the blot shows that at least some, if not all of the intact complex I contains NDUFA11 but nonetheless these normal complexes I are still not observed in SC, suggesting an indirect cause for this observation. The data may not be able to discriminate between possibilities A) and B) but please include a full discussion of this important aspect.
2. In the abstract it says “Their perturbation by this, or other means, is likely to be the cause of metabolic stress and disease”. This is probably a mistype, because this can’t be the only cause.
3. What do the terms “NDUFA11 activity’ and “nduf-11 activity’ mean? What activity does the subunit itself independently have?
4. In figure 4, the gels clearly show the effect on the amount of complex I and SC present, but the monomeric complex I band in both digitonin and tx100 appears slightly lower in mass in the cxI activity gels, but for the digitonin stained with commassie they appear at the same level. This was further confused by the term “lower molecular weight form of complex I” on page 8. Upon closer inspection, the junction between lanes for in-gel activity suggest that the gels were cut vertically, then reassembled manually before imaging – if that is the case please explicitly state this in the figure legend, or resection the images, like has been done for the commassie ones. When the samples are run in adjacent lanes, and before the gels are cut, are the bands at the same apparent molecular mass or not?
5. How were the respiratory complex bands in BN PAGE identified in this figure?

© 2021. Published by The Company of Biologists under the terms of the Creative Commons Attribution License (https://creativecommons.org/licenses/by/4.0/).

6. Mammalian NDUFA11 is around 15 kDa, but the C. elegans NDUF-11 is about double the size at 30 kDa. As the homology model shown in fig S1 only covers the homologous 4TMH bundle, could you perform secondary structure prediction to inform whether the N-terminal extension is likely to form for example additional transmembrane helices?
7. An explanation for loss of rcr in g/mal respiration appears to be that because there is less complex I in the kd, the enzyme population is working closer to its maximal rate, rather than being limited by membrane potential (unlike in the wt where membrane potential will be limiting the rate), such that when the membrane potential is dissipated, the kd g/mal rate does not increase by much, but the wt does. Similarly, the increased complex II content means that this rate is more limited by membrane potential in the kd, explaining a higher rcr. If this is the explanation being presented, could this be described a little more clearly in the text for a non-specialist to be able to follow?
8. On page 13 in the discussion, it says "This, in turn, allowed us to investigate how Complex I, when compromised in activity…Despite the reduction of Complex I activity". The phrasing is ambiguous; is the suggestion here that the individual complex I molecules have lower catalytic activity and compromised structure, or that you are assessing the effects of a lower content of fully-functional complex I?
9. In the lc-ms analyses, carbamidomethylation of cysteine was included as a modification, but the methods don't mention an iodoacetamide treatment - please clarify this.
10. For CryoET please state the slit width used for the GIF, and include microscope settings such as magnification, total dose and dose fractionation used.
11. For the hydrogen peroxide measurements, I am unclear what is meant by "plateau" or why the data are presented as Intensity in Fig 7g - Upon addition of substrates, the system should achieve a linear rate of resorufin production until substrates are exhausted, so a kinetic rate of H_2O_2 production can be calculated for each condition (or separately for burst and steady-state phases where applicable), using the resorufin extinction coefficient. It would be more informative to reanalyse the traces and present the rates of H_2O_2 production, rather than average signal intensities, and include example UV-vis traces for these measurements, like for the other data in this Figure.
12. Could a supplementary table be provided to accompany Fig S2 and Fig 3c to list the human homologue names for the C. elegans proteins labelled?

Reviewer 2

Advance summary and potential significance to field

This paper describes a detailed investigation of the consequence of defective NDUF-11 on respiratory fitness, mitochondrial ultra-structure, and overall organismal health in C. elegans. Mammalian NDUFA11 is known to function in the assembly of complex I, not to be involved in catalysis. This study identified and studied the worm homolog of NDUFA1, focusing on analyses of nduf-11 (RNAi) animals as the loss of B0491.5/nduf-11 in C. elegans causes developmental arrest at L2 larval stage. It was demonstrated that a significant reduction of B0491.5/NDUF-11 led to reduction of complex I and loss of its super-complexes which has consequences for mitochondrial health and organismal fitness.
Complex II-linked respiration is augmented likely as a compensatory mechanism.
The measurements of membrane potential and H_2O_2 production in isolated mitochondria are also consistent with alterations in complex I and II respiration. Proteome analyses revealed the downregulation of some other complex I subunits consistent with the destabilization of complex I and perturbations in glycolysis/ gluconeogenesis and fatty acid metabolism.
Looking into mitochondrial ultrastructure, morphological aberrations found in NDUF-11 depleted mitochondria include fragmented cristae, widening of cristae junction, and enlargement of intermembrane space.

---Whether these changes are specific to complex I or NDUF-11 defects is not studied.

Comments for the author

The essential findings in B0491.5/nduf-11 knockout and knockdown worms are in line with the previously known function of NDUFA11 and profound ETC enzyme deficiencies. Morphological and

© 2021. Published by The Company of Biologists under the terms of the Creative Commons Attribution License (https://creativecommons.org/licenses/by/4.0/).

proteomic analysis revealed novel interesting observations. Patients with NDUFA11 mutations have been reported, the dataset presented here is thus of value and scientific interest.
---However, I believe that the revision suggested below are ESSENTIAL to the potential value of the paper.

Specific comments and suggested revisions:
o It is not clear from the paper which developmental stage(s) of the worms that were used in the different assays. This needs to be specified and justified. Also, in general, liquid culture is known to be stressful to the worms. It is quite possible to collect enough worms to perform mitochondrial physiology experiments without liquid culture. Some such verification experiments should be done.
o The mitochondrial respiration measurement by Oxygraph was carried at 25°C. What is the reason for not doing it at 20°C which is the temperature used to culture the nematodes for the experiments? Also, the temperatures at which the other assays with isolated mitochondria (TMRM and Amplex Red assays)
were carried out should be reported.
o I can't agree with the authors' discussion and conclusions about the mitochondrial respiratory competency of nduf-11 (RNAi) animals. To my knowledge, the baseline respiration of isolated mitochondria, that is oxygen consumption in the presence of a substrate but before the addition of ADP only exists in vitro. It represents utilization of the substrate and is an indicator of leak respiration. Part of it is due to the presence of damaged mitochondria in the preparation. Therefore, the finding of similar rates of baseline respiration in the presence of pyruvate/malate for the mitochondria with native or depleted levels of NDUF-11 cannot be interpreted to indicate that the mutant mitochondria are respiration-competent. Instead, overall, the data of state 3 rates (ADP-stimulated) indicate impaired respiratory function after decreasing the level of NDUF-11 expression. In this regard, one straightforward experiment to further investigate the effect on bioenergetic fitness is to measure ATP levels. Which should be done.
o On page 13, the authors state "25% to 75% increase in expression of Complex II subunits", but there is no evidence presented. Please add this data. The authors also state that the upregulation of complex II could underlie enhanced complex II respiration. Could the authors measure the activity of complex II? A higher activity would strengthen the statements and result of upregulated complex II subunits.
o A higher complex II activity could strongly suggest that the compensatory increase of complex II-linked respiration occurs via the upregulation of complex II subunits and activity.
o The citations of Fig. 6J and 6I are incorrect.
o Please cite Fig. 7i in the manuscript text.
o Fig. 7C: "succ." Should be added to the substrate label in the OCR trace graph.

First revision

Author response to reviewers' comments

Dear Prof Way, and reviewers

Many thanks for giving us the opportunity to submit a revised version of our manuscript *'Maintenance of Complex I and its super-complexes by NDUF-11 is essential for mitochondrial structure, function and health'*. We are very grateful to you and the referees for your thorough evaluation of the work and constructive feedback. Indeed, their suggestions were discerning and genuinely helpful. Their insight inspired new experiments, which has confirmed one of our original interpretations. Thus, the revised manuscript includes a new result, resolving a major concern of one of the reviewers, while further queries have been addressed through amendments to the manuscript, and in the response below. We have also generally improved the text through further editing and refinement.

The new submission we believe settles the issues raised by the review, and clarifies those points that were unclear in the initial version. We hope you will agree that the new

© 2021. Published by The Company of Biologists under the terms of the Creative Commons Attribution License (https://creativecommons.org/licenses/by/4.0/).

manuscript is greatly improved by the changes, and is now suitable for publication in JOCS. We thank you all for help in achieving this.

A point-by-point response to the editorial and referees' comments is included below. Yours Sincerely,

Ian Collinson

Editor
The length limit for Research Articles is 8000 words, and 3000 for Short Reports. This word count does not include the title page, abstract, materials & methods, or reference list but does include figure legends.

We have checked, and the manuscript has 6,948 words excluding the sections mentioned above, and have added this information to the title page.

Please note that the original source files for text, tables and all figures will be required. Do not upload a single pdf containing tables and figures but instead provide a separate file for each item (i.e. a Word file containing the main text, tables and legends, and a separate file for each figure - see here for required file formats).

The figure files are now submitted as separated files. Their legends are included with the main text, which also includes the tables and figure legends for the supplementary material.

Supplementary material may include movies, figures and tables. Please collate supplementary figures and tables into a single PDF file. If your tables are very large, or you wish readers to be able to export and/or manipulate the data, we would prefer you to submit them as Microsoft Excel files. Movies and cover image suggestions can be uploaded separately.

Supplementary figures are now collated in a single supplementary file/pdf. Movies are provided in individual files.

We ask for no more than eight display items (figures, tables, etc.), each fitting up to a page, to be included with the manuscript. Since you currently have more than the permitted number of display items, please can you reduce the number of display items you have.

Main Table 1 and 2 have been moved to the Supplementary Material and we now include a new Figure regarding Complex II activity. Still, the total display items are 8 and thus within the allowed limit.

We ask for supplementary figures to be submitted as one PDF file, including figure legends. As your supplementary figures are currently in your main manuscript file, we would appreciate it if you could separate these into one PDF.

All Supplementary Figures and Tables are now submitted in a single PDF alongside their respective captions.

Please see our website for further guidelines on supplementary material.

We are now requesting that authors fill in and upload a submission checklist with their manuscript. This form asks authors to confirm that they have followed best practice guidelines regarding experimental subjects, data reporting and statistics. The checklist is based on the NIH Principles and Guidelines for Reporting Preclinical Research and is intended to help ensure high standards for reporting and to aid reproducibility.
Please fill in and upload the submission checklist into the supplemental files area. You can find a copy of the checklist here.
We have filled the required form and have submitted it during the revision process.
In regards to the antibodies used in this study, we have not performed validation of commercial antibodies, and there is no validation available at the moment, but they have been used

© 2021. Published by The Company of Biologists under the terms of the Creative Commons Attribution License (https://creativecommons.org/licenses/by/4.0/).

successfully before: (ab14711) Anti-NDUFS3 antibody [17D95] in PMID: 20971856; and, (ab14748) Anti-ATP5A antibody [15H4C4]in PMID: 20584279. All other relevant information is available in the Material and Methods section.

In regards to Figures with sample size n<5, although we have used n=3 mitochondria, within this a number of crista/CJs/IMS widths were averaged. Mean crista SA/volume, IMS width and CJ diameter for Figures 6 and S3 were calculated from: N=3 mitochondria for each condition, with n=37 (wild-type) and 105 (NDUF-11) cristae analysed; N=3 mitochondria for each condition, n=4 points per mitochondrion analysed; and, N=3 mitochondria for each condition, with n=37 (wild-type) and n=55 (NDUF-11) CJ analysed, respectively. This has been added to the corresponding section on Material and Methods.

To help you meet your funder requirements to report all financial support received, we require authors to provide details of all their funding sources during submission.

To add these details, go to: http://submit-jcs.biologists.org, enter your 'Author Area' and click on the queue 'Papers Returned for Your Attention'.

Click on the section ‘Funding Information’, where you will see instructions on how to enter your funder names and grant numbers.

The funding data submitted should match the information provided in the paper's Funding section. Please provide funder names in full.

The above requirements have been addressed during the submission of revisions.

Reviewer 1
Advance Summary and Potential Significance to Field:
Knapp-Wilson et al describes a C. elegans model for the disturbance of complex I and supercomplex assembly and/or their structural integrity. The authors identify the C. elegans homologue for subunit NDUFA11, which structurally forms contact sites between complexes I and III in mitochondrial respiratory supercomplexes. The gene knockout of this subunit was lethal, but a strong knockdown was viable and investigated here. The content of intact complex I was much reduced, and there appear to be no stable complex I-containing supercomplexes present. The consequences of these primary effects are on mitochondrial coupling, striking changes to mitochondrial morphology, and a series of metabolic compensatory shifts. Despite these mitochondrial and metabolic perturbations, the animals experienced a longer lifespan than their WT counterparts, but their progeny failed to develop to maturity, indicating a lack of metabolic plasticity under high mitochondrial demands.
We thank the Reviewer for their clear summary of our paper.
This study represents a well-designed and careful body of work that advances knowledge of the effects of complex I and SC dysfunction on mitochondria and on animal health, presented clearly and discussed in an unbiased manner.

We are pleased that the Reviewer considers our work of value.

Reviewer 1 Comments for the Author:
1. Could the authors comment on whether NDUF-11 is essential, or just beneficial for complex I to otherwise reach maturity (with the flavoprotein domain attached)?

Mammalian NDUFA-11 is a supernumerary subunit and is considered to be an assembly factor for Complex I. Our data and experimental design do not discriminate between the different assembly modules of Complex I, in part because we lack immunoblot detection in native conditions. However, if one is allowed to extrapolate from reports in mammalian samples, NDUFA-11 is known to be important for the addition of the distal components ND-4/5 to the membrane arm of Complex I (PMC: 3839705). Under this modular assembly, the matrix-facing N-module comprising the flavoprotein and catalytic site of Complex I is the last addition to the complex. Therefore, one would consider NDUF-11 to be essential for Complex I maturation, in both worms and mammals, and presumably everything in between.

© 2021. Published by The Company of Biologists under the terms of the Creative Commons Attribution License (https://creativecommons.org/licenses/by/4.0/).

The possibilities in the knockdown are A) all of the intact cxI observed has the residual NDUF-11, but the quantity of mature complex I is limited to the supply of NDUF-11 B) some of the complex I observed does not have NDUF-11 but otherwise reaches maturity and could therefore be catalytically and structurally compromised or C) none of the mature complex I contains NDUF-11. As the g/m/adp rate of oxygen consumption looks to be ~ 20% of WT, this is consistent with option A) because as long as the catalytic rate limiting step has not changed, it should be proportional to the amount of complex I present. On the other hand, it is not clear from the monomeric complex I observed on in-gel activity on the BN PAGE whether or not the mass has shifted (see below). The lack of SC in digitonin does not seem to inform on this because the blot shows that at least some, if not all of the intact complex I contains NDUFA11 but nonetheless these normal complexes I are still not observed in SC, suggesting an indirect cause for this observation. The data may not be able to discriminate between possibilities A) and B) but please include a full discussion of this important aspect.

These are interesting and very important questions raised by the Reviewer. As mentioned above, our experimental design can only identify competent Complex I and derived structures of the mature complex but is not able to detect sub-assembly stages. Considering the described role of NDUFA-11 in Complex I assembly, our data suggests that the residual NDUF-11 is present in the observed (mature) Complex I and, as mentioned by the Reviewer, the total levels of the complex is limited by the supply of NDUF-11. Only fully assembled and mature Complex I will have catalytic activity, since the N-module is the last to be added to the complex. Therefore, all the observed Complex I-positive bands in our studies correspond to mature Complex I containing NDUF-11 because from the 2D BN- PAGE the NDUF-11 immunoblot signal is observed in all Complex I-positive bands. Words to this effect have been added to the discussion.

2. In the abstract it says “Their perturbation by this, or other means, is likely to be the cause of metabolic stress and disease”. This is probably a mistype, because this can’t be the only cause.

We amended the last sentence of the abstract to remove this misleading statement.

3. What do the terms “NDUFA11 activity’ and “nduf-11 activity’ mean? What activity does the subunit itself independently have?

NDUFA11 and nduf-11 are merely structural proteins and contain no known (enzymatic) activity. The term ‘activity’ has now been changed to (protein) ‘levels’.

4. In figure 4, the gels clearly show the effect on the amount of complex I and SC present, but the monomeric complex I band in both digitonin and tx100 appears slightly lower in mass in the cxI activity gels, but for the digitonin stained with commassie they appear at the same level. This was further confused by the term “lower molecular weight form of complex I” on page 8. Upon closer inspection, the junction between lanes for in-gel activity suggest that the gels were cut vertically, then reassembled manually before imaging – if that is the case please explicitly state this in the figure legend, or resection the images, like has been done for the commassie ones. When the samples are run in adjacent lanes, and before the gels are cut, are the bands at the same apparent molecular mass or not?

The samples shown in Fig. 4 were ran in the same gel/ conditions, but the lanes were cut out vertically so samples/lanes in the same gel could be processed for different assays/ stains/ 2D. The Reviewer is right that the original images were misleading possibly because of the manual alignment done prior to the image acquisition. Therefore, we now state in the figure legend that the lanes were cut vertically and then reassembled prior to imaging, as suggested. We also provide uncropped images of the gel lanes (Coomassie and CI activity) in the Supp. Fig. S3. Additionally, we also made it more evident in the figure that the lanes were not adjacent (white bar separation).

We also changed the sentence highlighted by the Reviewer to the following, which we believe will be clearer to the reader:

‘Conversely, NDUF-11-depleted samples contain only the **lower molecular**

© 2021. Published by The Company of Biologists under the terms of the Creative Commons Attribution License (https://creativecommons.org/licenses/by/4.0/).

> **weight form of Complex I-positive super-complexes** (Fig. 4c; asterisk), and critically are devoid of higher molecular weight super-complexes seen in the control.'

Supp. Fig. S3 includes representative gels were Control and nduf-11_RNAi samples were run in adjacent lanes. Although these gels are unstained, one can easily observe that the band pattern is identical between groups, with the exception of the high order forms of super-complexes, which disappear in nduf-11_RNAi group. We also provide representative images of Control samples treated with Triton X-100 *vs* Digitonin ran in adjacent lanes for the Reviewer and reader inspection.
Overall, the migration pattern of Complex I in Triton X-100 treated mitochondria observed by the in- gel activity is identical between groups. We attribute the small nuances in shift to the running conditions experienced by the samples in those lanes rather than a true biological effect. However, in regards to the digitonin-treated samples there is clearly a faint band of low molecular weight that is not observed in the control group. Therefore, we are confident that the panels in main figure 3 are representative of the siRNA treatment and that our interpretation and discussion are valid. The following was added to the main text:

> 'Interestingly, NDUF-11-depleted samples show a faint band below the super-complexes which is not observed in the N2 control group (Fig. 4c; arrowhead). Importantly, the migration pattern of this band is significantly different from the monomeric form of Complex I observed in samples extracted by TX-100 (Fig. 4c *vs* Fig. 4a and Fig. S3h). Although the apparent migration pattern of the monomers can be different depending on the type of detergent used, it should be noted that the worm mitochondrial digitonin extracts have negligible to no levels of monomeric Complex I, compared to extractions of bovine mitochondria (Fig. S5b). Interestingly, the lower molecular weight Complex I-positive super-complex (*i.e.* the faint band highlighted by the arrowhead on Fig. 4) resembles that present in bovine mitochondria (Fig. S5b white arrowhead), suggesting that it could be the SC I:III2 core unit. Both observations have been previously reported in Complex IV-deficient worms [26].'

5. *How were the respiratory complex bands in BN PAGE identified in this figure?*

The identification was done by in-gel activities for Complex I, IV and V using standard biochemical assays for this purpose. We are now providing representative images in Supp. Fig. S5 for control samples treated with Triton X-100 or Digitonin and, we have changed the figure legend on main Fig. 4 from 'data not shown' to 'see Supp. Fig. S5'.

Additionally, we also provide a side-by-side comparison of worm mitochondria *vs* bovine mitochondria in Supp. Fig. 5 to highlight the different super-complex organisation in both organisms. This allowed us to state with confidence that under physiological conditions the levels of monomeric Complex I in worm mitochondria are only vestigial to none.

6. *Mammalian NDUFA11 is around 15 kDa, but the C. elegans NDUF-11 is about double the size at 30 kDa. As the homology model shown in fig S1 only covers the homologous 4TMH bundle, could you perform secondary structure prediction to inform whether the N-terminal extension is likely to form for example additional transmembrane helices?*

The Reviewer raises a valid question. In our homology model we did not model the N-terminus (155 aa; 17.5 kDa) and the C-terminus (37 aa; 4 kDa) as these regions have very low predicted secondary structure, and no predicted TM regions (see image below). This means that they are likely extra- membrane loop regions, therefore effectively impossible to model with any certainty, hence we ignored them. Even discounting this, the purpose of the modelling and MD in the present study was to support the identification of the *C. elegans* protein as a NDUFA11 homologue - a task achieved by showing the main part of the protein has structural homology to the mammalian protein. Phyre2, a free web-based service for protein structure prediction, also assigns the N-terminus one short non-TM helix from residue ~100 and everything else is loop with no homology to any known domains.

© 2021. Published by The Company of Biologists under the terms of the Creative Commons Attribution License (https://creativecommons.org/licenses/by/4.0/).

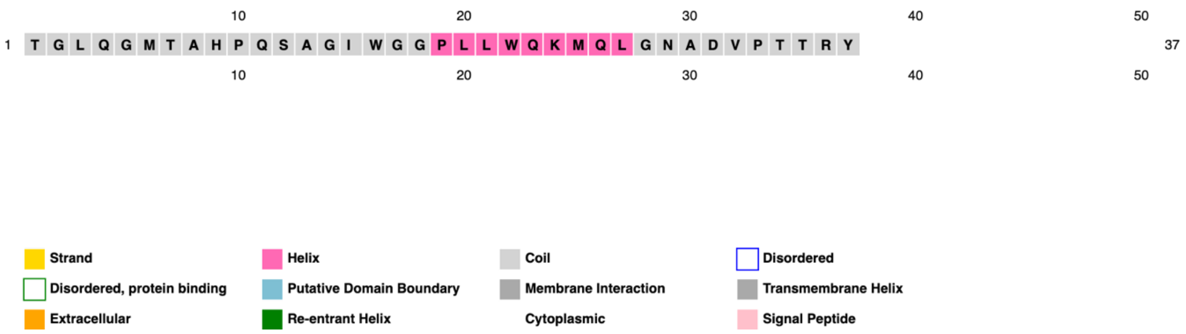

7. An explanation for loss of rcr in g/mal respiration appears to be that because there is less complex I in the kd, the enzyme population is working closer to its maximal rate, rather than being limited by membrane potential (unlike in the wt where membrane potential will be limiting the rate), such that when the membrane potential is dissipated, the kd g/mal rate does not increase by much, but the wt does. Similarly, the increased complex II content means that this rate is more limited by membrane potential in the kd, explaining a higher rcr. If this is the explanation being presented, could this be described a little more clearly in the text for a non-specialist to be able to follow?

The reduced respiratory control ratio seen with pyruvate malate in the knockdown mitochondria reflects the low activity of complex I (new data on Fig. 8, and associated text in the results section). While this is sufficient to maintain rates of respiration in State 4, where the proton motive force restricts electron flow, this is not the case in State 3 where the reduced proton motive force allows complex I to operate close to its maximal rate. Conversely, with succinate as substrate, the increased activity of complex II has little effect on the State 4 rate of respiration but increases State 3 respiration leading to an increase in respiratory control ratio.

8. On page 13 in the discussion, it says “This, in turn, allowed us to investigate how Complex I, when compromised in activity…Despite the reduction of Complex I activity”. The phrasing is ambiguous; is the suggestion here that the individual complex I molecules have lower catalytic activity and compromised structure, or that you are assessing the effects of a lower content of fully-functional complex I?

We removed the word ‘activity’ from the sentence since we have not measured Complex I activity directly. We also changed the follow up sentence to ‘Despite the major loss of Complex I and its ability to […]’.

Our experimental setup cannot discriminate catalytic activity of particular assembled entities levels. Instead, our data seems to suggest that the residual levels of NDUF-11 are sufficient to assemble a competent and functional Complex I and integrate it in super-complexes. Words to this effect have been added to the text.

9. In the lc-ms analyses, carbamidomethylation of cysteine was included as a modification, but the methods don’t mention an iodoacetamide treatment - please clarify this.

Correct, we did not conduct an iodoacetamide treatment. Most likely, these cysteine modification modifications occurred at some point during sample preparation – as is common with the reactive thiol of cysteines. A note was added to the text stating this.

10. For CryoET please state the slit width used for the GIF, and include microscope settings such as magnification, total dose and dose fractionation used.

The required information has been added to the text. Briefly:

© 2021. Published by The Company of Biologists under the terms of the Creative Commons Attribution License (https://creativecommons.org/licenses/by/4.0/).

'Cryo-ET was performed using a Talos Arctica, equipped with a 200 kV FEG (Thermo Fisher Scientific), K2 DED and GIF BioQuantum energy filter **operated with a slit width of 20eV** (Gatan, Pleasanton, USA). Dose-fractionated tomograms **(2-5 frames per tilt)** were collected **at a magnification of 49kx (corresponding to a pixel size of 2.8 Å),** typically collected from +60° to 60° with tilt steps of two degrees. **The total dose per tomogram was <120 e^-/ Å^2.**'

11. For the hydrogen peroxide measurements, I am unclear what is meant by "plateau" or why the data are presented as Intensity in Fig 7g - Upon addition of substrates, the system should achieve a linear rate of resorufin production until substrates are exhausted, so a kinetic rate of H2O2 production can be calculated for each condition (or separately for burst and steady-state phases where applicable), using the resorufin extinction coefficient. It would be more informative to reanalyse the traces and present the rates of H2O2 production, rather than average signal intensities, and include example UV-vis traces for these measurements, like for the other data in this Figure.

We now provide representative traces for the Amplex Red experiments, as requested. As can be seen in the new panels of Fig. 7, the traces are linear throughout the experimental window and reach a linear rate after the addition of the different substrates/inhibitors, which are not exhausted. We apologise for the confusion that the initial text created my mentioning a 'plateau; this was a typo from copying part of the information from the TMRM section. The Amplex Red methods section has been amended to show the correct description about the method and data analysis.

Although the Reviewer is correct in that the ideal approach to analyse the data is to extract the linear rates and convert it to absolute values, we would argue that considering we were running samples in parallel under the same experimental conditions and timeframes, choosing a specific time point for data extraction is still valid. Moreover, since we are interested in relative changes rather than absolute rates, showing relative units are also appropriate. Still, we are now showing the data on Fig. 7h as percentage to substrate-only values, which we believe might create less confusion to the reader. The main text has been updated and we lowered the importance given to the ROS effects which are clearly mild and would only be experience under stress conditions *in vivo*, considering the obtained data in our experimental setup with isolated mitochondrial fractions.

12. Could a supplementary table be provided to accompany Fig S2 and Fig 3c to list the human homologue names for the C. elegans proteins labelled?

We now provide a supplementary table (S2) where the highlighted proteins on Fig. S2 and 3C are shown alongside their human orthologues. The citation to this information has been added to the respective figure legends.

Reviewer 2
<u>*Advance Summary and Potential Significance to Field:*</u>
This paper describes a detailed investigation of the consequence of defective NDUF-11 on respiratory fitness, mitochondrial ultra-structure, and overall organismal health in C. elegans. Mammalian NDUFA11 is known to function in the assembly of complex I, not to be involved in catalysis. This study identified and studied the worm homolog of NDUFA1, focusing on analyses of nduf-11 (RNAi) animals as the loss of B0491.5/nduf-11 in C. elegans causes developmental arrest at L2 larval stage.
It was demonstrated that a significant reduction of B0491.5/NDUF-11 led to reduction of complex I and loss of its super-complexes which has consequences for mitochondrial health and organismal fitness. Complex II-linked respiration is augmented likely as a compensatory mechanism.
The measurements of membrane potential and H2O2 production in isolated mitochondria are also consistent with alterations in complex I and II respiration. Proteome analyses revealed the downregulation of some other complex I subunits consistent with the destabilization of complex I and perturbations in glycolysis/ gluconeogenesis and fatty acid metabolism.

© 2021. Published by The Company of Biologists under the terms of the Creative Commons Attribution License (https://creativecommons.org/licenses/by/4.0/).

Looking into mitochondrial ultrastructure, morphological aberrations found in NDUF-11 depleted mitochondria include fragmented cristae, widening of cristae junction, and enlargement of intermembrane space.

We thank the Reviewer for their clear summary of our paper.

---Whether these changes are specific to complex I or NDUF-11 defects is not studied.

Reviewer 2 Comments for the Author:
The essential findings in B0491.5/nduf-11 knockout and knockdown worms are in line with the previously known function of NDUFA11 and profound ETC enzyme deficiencies. Morphological and proteomic analysis revealed novel interesting observations. Patients with NDUFA11 mutations have been reported, the dataset presented here is thus of value and scientific interest.

We are pleased that the Reviewer considers our work of value.

---However, I believe that the revision suggested below are ESSENTIAL to the potential value of the paper.

Specific comments and suggested revisions:
1) It is not clear from the paper which developmental stage(s) of the worms that were used in the different assays. This needs to be specified and justified. Also, in general, liquid culture is known to be stressful to the worms. It is quite possible to collect enough worms to perform mitochondrial physiology experiments without liquid culture. Some such verification experiments should be done.

All data shown in the manuscript was collected from adult worms, except that shown on Table S1 (brood count) which represents worms at different developmental levels. We are now including a new sentence in the Material and Methods to alert the reader for this.
We are aware of the differences between plate and liquid cultures; however, we had to switch to the latter because otherwise we could not obtain enough biological sample (isolated mitochondrial fraction) to carry all the biochemical assays on directly comparative sample, namely, BN-PAGE, O2 consumption, TMRM assay, Amplex Red assay and, Complex II activity (new data).

2) The mitochondrial respiration measurement by Oxygraph was carried at 25°C. What is the reason for not doing it at 20°C which is the temperature used to culture the nematodes for the experiments? Also, the temperatures at which the other assays with isolated mitochondria (TMRM and Amplex Red assays) were carried out should be reported.

We followed the protocol from Paul S Brooke’s Lab (Andrew P. Wojtovich, personal communication) that has been previously published (PMID: 18809388), where mitochondrial assays were carried at 25°C. Reference to this work was already in the text but now we include it next to the buffer composition and temperature used for the O2 consumption assays.

The TMRM and Amplex Red experiments were carried at room temperature since the spectrophotometer used was not coupled to a water-bath/temperature control unit. However, since room temperature in our building is within 18-22 °C we considered this to be withing the physiological range for *C. elegans*. We have previously stated that TMRM measurements were caried at room temperature but we have now added the same information to the Amplex Red section.

3) I can’t agree with the authors’ discussion and conclusions about the mitochondrial respiratory competency of nduf-11 (RNAi) animals. To my knowledge, the baseline respiration of isolated mitochondria, that is oxygen consumption in the presence of a substrate but before the addition of ADP, only exists in vitro. It represents utilization of the substrate and is an indicator of leak respiration. Part of it is due to the presence of damaged mitochondria in the preparation. Therefore, the finding of similar rates of baseline respiration in the presence of pyruvate/malate for the mitochondria with native or depleted levels of NDUF-11 cannot be interpreted to indicate

© 2021. Published by The Company of Biologists under the terms of the Creative Commons Attribution License (https://creativecommons.org/licenses/by/4.0/).

that the mutant mitochondria are respiration-competent. Instead, overall, the data of state 3 rates (ADP-stimulated) indicate impaired respiratory function after decreasing the level of NDUF-11 expression. In this regard, one straightforward experiment to further investigate the effect on bioenergetic fitness is to measure ATP levels. Which should be done.

We appreciate the point being made by the Reviewer but believe that the higher respiratory control ratio seen in data with succinate in the NDUF-11 knockdown mitochondria provide strong evidence that they are respiration competent and not damaged. Thus, the data of Fig. 7b show that with succinate as substrate there is a strong ADP stimulation which is reversed by oligomycin and then reinstated with uncoupler. This is strong evidence for well coupled mitochondria - almost a definition - and thus we do not think that the ATP measurements suggested by the Reviewer are required. Note also that point 7 of Reviewer #1 would appear to agree with our conclusion and in response to their request we have amplified the text to make the point clearer.

4) On page 13, the authors state "25% to 75% increase in expression of Complex II subunits", but there is no evidence presented. Please add this data. The authors also state that the upregulation of complex II could underlie enhanced complex II respiration. Could the authors measure the activity of complex II? A higher activity would strengthen the statements and result of upregulated complex II subunits.

This data was initially presented in the volcano plots of Fig. S2. However, we now state the fold- change of the four Complex II subunits in the Results section. The following sentence was added in line with the text:

> 'Crucially, for the present investigation (see also below), the four subunits of Complex II, SDHA-1, SDHB, MEV-1 and SDHD-1 (Fig. S2), were upregulated by 1.73 (p=0.0225), 1.39 (p=0.0188), 1.24 (p=0.1209) and 1.28 (p=0.009) fold, respectively.'

In regards to the Complex II activity, please see the reply to point 5 below.

5) A higher complex II activity could strongly suggest that the compensatory increase of complex II- linked respiration occurs via the upregulation of complex II subunits and activity.

We have measured Complex II (succinate dehydrogenase) activity and well as Complex I (NADH dehydrogenase activity) in isolated mitochondrial fractions from N2 controls and nduf-11_RNAi animals.

The data confirms that Complex II activity is partially increased and Complex I activity is significantly decreased in the RNAi group compared to controls. This is in line with the proteomic data showing a mild upregulation of Complex II and downregulation of Complex I. Considering the nominal increase in Complex II values for the proteomic and biochemical data, we still consider that the best explanation for the observed increase in oxygen consumption experienced by NDUF-11 depleted mitochondria when energised with exogenous succinate is due to the low levels of oxaloacetate, a strong Complex II inhibitor. Obviously, the minimal increase in Complex II levels/ activity will synergise with this factor.

The data is presented in the new Fig. 8 and the main text and discussion have been updated accordingly.

6) The citations of Fig. 6J and 6I are incorrect.
We thank the Reviewer for pointing this out. The panel id on Figure 6 has been updated as well as its caption. The citation of Figure 6 in the main text, namely, for panel i-l has been amended.

7) Please cite Fig. 7i in the manuscript text.

This has been corrected. We now cite panel i of Fig. 7 and briefly describe its associated data in the text.

8) Fig. 7C: "succ." Should be added to the substrate label in the OCR trace graph.

© 2021. Published by The Company of Biologists under the terms of the Creative Commons Attribution License (https://creativecommons.org/licenses/by/4.0/).

Thank you for pointing this out. We have added the 'succ' abbreviation to the list abbreviation shown at the end of Fig. 7 legend.

Second decision letter

MS ID#: JOCES/2021/258399

MS TITLE: Maintenance of Complex I and its super-complexes by NDUF-11 is essential for mitochondrial structure, function and health

AUTHORS: Amber Knapp-Wilson, Goncalo C Pereira, Emma Buzzard, Holly C Ford, Andrew P Richardson, Robin A Corey, Chris Neal, Paul Verkade, Andrew Halestrap, Vicki AM Gold, Patricia E Kuwabara, and Ian Collinson
ARTICLE TYPE: Research Article

We have now reached a decision on the above manuscript.

To see the reviewers' reports and a copy of this decision letter, please go to: https://submit-jcs.biologists.org and click on the 'Manuscripts with Decisions' queue in the Author Area. (Corresponding author only has access to reviews.)

As you will see, both reviewers gave favourable reports and support publication although they have raised some points. Reviewer 1 has raised a minor point that will require amendments to your manuscript. Reviewer 2 feels that two previous concerns have not been addressed. I hope that you will be able to carry these out because I would like to be able to accept your paper.

We are aware that you may be experiencing disruption to the normal running of your lab that makes experimental revisions challenging. If it would be helpful, we encourage you to contact us to discuss your revision in greater detail. Please send us a point-by-point response indicating where you are able to address concerns raised (either experimentally or by changes to the text) and where you will not be able to do so within the normal timeframe of a revision. We will then provide further guidance. Please also note that we are happy to extend revision timeframes as necessary.

Please ensure that you clearly highlight all changes made in the revised manuscript. Please avoid using 'Tracked changes' in Word files as these are lost in PDF conversion.

I should be grateful if you would also provide a point-by-point response detailing how you have dealt with the points raised by the reviewers in the 'Response to Reviewers' box. Please attend to all of the reviewers' comments. If you do not agree with any of their criticisms or suggestions please explain clearly why this is so.

Reviewer 1

Advance summary and potential significance to field

I thank the authors for carefully addressing my comments, they have now presented an improved version of this work.

Comments for the author

I have one very minor further request prior to publication: Please include the Phyre2 prediction result for the unmodelled nduf-11 N-terminus, similar to what is described in the author response, in the manuscript.

© 2021. Published by The Company of Biologists under the terms of the Creative Commons Attribution License (https://creativecommons.org/licenses/by/4.0/).

Reviewer 2

Advance summary and potential significance to field

In their revisions, the authors address well many of the reviewers' suggestions.

However, I still have the following comments:

- The authors argue that their nduf-11(RNAi) worms are "respiration-competent" because the RCR of succinate-mediated respiration is well coupled.

Yet they show that in the nduf-11 knockdown mitochondria, succinate does not fully rescue the ADP-sustained respiration when added after Complex I-linked substrates pyruvate/malate. It would have been good to measure and compare the oxygen consumption of nduf-1 knock-down and wild type worms.

- I suggested to measure ATP levels. Their response is “not required”.

This is just a little surprising because: 1) why is it not required? 2) making ATP is what the electron transport chain is for, 3) measuring ATP is a standard technique for which one uses a kit (although one must be careful with ATP extraction).

Comments for the author

- It is well appreciated that the authors have made serious efforts to answer both reviewers' comments. I don't completely understand why the points I mention now in the Comments for the authors have not been addressed. I suggest that it should be suggested to the authors to address them. But I don't think the paper should be held up if they don't, don't want to, or can't.

Second revision

Author response to reviewers' comments

Reviewer 1 Advance Summary and Potential Significance to Field:

I thank the authors for carefully addressing my comments, they have now presented an improved version of this work.

Reviewer 1 Comments for the Author:

I have one very minor further request prior to publication: Please include the Phyre2 prediction result for the unmodelled nduf-11 N-terminus, similar to what is described in the author response, in the manuscript.

We have included the following statement at the end of the modelling section in the main text:

> “As mentioned above, B0491.5 has a considerable (155 aa) N-terminus; however, we omitted both the N- and C-termini from our homology model as these regions have very low predicted secondary structure, and no predicted TM regions, suggesting that they are likely extra-membrane loop regions

We are reluctant to extend the discussion to include the Phyre2 prediction result, as the output is considered “highly speculative” by Phyre2. If we model the N-terminal 155 residues, the prediction algorithm is unable to assign anything to the first 124 residues (i.e. probably no structure), and the rest are modelled with a dismal 25.5% confidence as a 13 residue helix followed by a random coil.

Overall, the Phyre2 is unable to successfully model anything for the bulk of the N-terminus. The reason we have included it in our last round of revisions was simply for the Reviewer perusal and to support our rebuttal and we never intended to include it in the main text.

© 2021. Published by The Company of Biologists under the terms of the Creative Commons Attribution License (https://creativecommons.org/licenses/by/4.0/).

Reviewer 2 Advance Summary and Potential Significance to Field:
In their revisions, the authors address well many of the reviewers' suggestions.

However, I still have the following comments:

•The authors argue that their nduf-11(RNAi) worms are 'respiration- competent" because the RCR of succinate-mediated respiration is well coupled.
Yet they show that in the nduf-11 knockdown mitochondria, succinate does not fully rescue the ADP-sustained respiration when added after Complex I-linked substrates pyruvate/malate. It would have been good to measure and compare the oxygen consumption of nduf-1 knock-down and wild type worms.

Our original submission already addressed this question regarding the 'partial' rescue by succinate when added after pyruvate/malate. We quote below the original text from the discussion:

> "Interestingly, succinate does not fully rescue the ADP-sustained respiration when added after the Complex I-linked substrates pyruvate/ malate. Under these experimental conditions, the residual levels of Complex I in NDUF-11 depleted mitochondria are insufficient to support maximal rates of respiration and a greater contribution from succinate is expected. However, the residual Complex I activity will lead to the production of some oxaloacetate which is an extremely potent inhibitor of Complex II (Stepanova et al., 2016; Zeyelmaker and Slater, 1967). This will reduce rates of succinate oxidation relative to native levels and so lower the maximal respiration as observed. Although the same inhibitory effect of oxaloacetate on Complex II activity would be experienced by wild-type mitochondria, the higher levels of Complex I in this group can fully sustain maximal rates of respiration and therefore the contribution required from Complex II is minimal."

•I suggested to measure ATP levels. Their response is "not required".
This is just a little surprising because: 1) why is it not required? 2) making ATP is what the electron transport chain is for, 3) measuring ATP is a standard technique for which one uses a kit (although one must be careful with ATP extraction).

Sorry, we should have said "not essential for the overall conclusions of the study".

The point made by the reviewer is very reasonable. We may well expect the ATP levels of the whole worms to be lower if they have reduced CI activity because succinate alone cannot drive respiration *in vivo* where it is made *via* the citric acid cycle whose operation generates NADH and thus requires CI. However, whole worm ATP production will reflect a balance of glycolysis and ox/phos and only in those tissues that are highly dependent the latter would the ATP levels drop appreciably. Thus, in this context, measuring whole worm ATP levels might not be very revealing.

Of course, in an ideal world we would still do it. But, from a pragmatic point of view it would considerably delay the paper. We would need to make more RNAi worms, and produce robust extraction protocols and measurements of ATP, which would not be quick (we are not big fans of kits with proprietary/ undisclosed compositions).

Hopefully, you will agree that this point is not a deal breaker. We have added an additional sentence to the discussion to spell out the point of depleted levels of ATP.

Reviewer 2 Comments for the Author:
•It is well appreciated that the authors have made serious efforts to answer both reviewers' comments. I don't completely understand why the points I mention now in the comments for the authors have not been addressed. I suggest that it should be suggested to the authors to address them.

See above. The issue is timeliness; I believe what would be gained from suggested additional experiments would be outweighed by the considerable delay this would cause to publication

© 2021. Published by The Company of Biologists under the terms of the Creative Commons Attribution License (https://creativecommons.org/licenses/by/4.0/).

But I don't think the paper should be held up if they don't, don't want to, or can't.

We certainly agree with that! But, of course it is "can't" rather than "don't want to".

Third decision letter

MS ID#: JOCES/2021/258399

MS TITLE: Maintenance of Complex I and its super-complexes by NDUF-11 is essential for mitochondrial structure, function and health

AUTHORS: Amber Knapp-Wilson, Goncalo C Pereira, Emma Buzzard, Holly C Ford, Andrew P Richardson, Robin A Corey, Chris Neal, Paul Verkade, Andrew Halestrap, Vicki AM Gold, Patricia E Kuwabara, and Ian Collinson
ARTICLE TYPE: Research Article

I am happy to tell you that your manuscript has been accepted for publication in Journal of Cell Science, pending standard ethics checks.

© 2021. Published by The Company of Biologists under the terms of the Creative Commons Attribution License (https://creativecommons.org/licenses/by/4.0/).
